# Determinants of and interventions for Proton Pump Inhibitor prescription behavior: A systematic scoping review

**DOI:** 10.1186/s12875-024-02459-5

**Published:** 2024-06-11

**Authors:** L. C. van Gestel, M. A. Adriaanse, S. L Kanis, S. M. Mensink-Bout, J. W. Schoones, M. E. Numans, J. C. Kiefte-de Jong, G. van den Brink

**Affiliations:** 1 Health, Medical and Neuropsychology Unit, Leiden University, Leiden, The Netherlands; 2 Department of Public Health and Primary Care, Leiden University Medical Center, Leiden, The Netherlands; 3 Directorate of Research Policy, Leiden University Medical Center, Leiden, The Netherlands

**Keywords:** Proton pump inhibitors, Prescribing behavior, Behavioral determinants, Behaviour Change Wheel

## Abstract

**Background:**

Proton Pump Inhibitors (PPI) are frequently prescribed. Long-term use is associated with side-effects and patients often lack a valid indication. Inappropriate PPI prescribing thus needs to be addressed. This review aims to scope 1) what determinants are studied as reasons for PPI prescribing, 2) what strategies are used for changing PPI (de)prescribing, and 3) whether important determinants are addressed in these interventions.

**Methods:**

We searched eight databases for papers on determinants of physician PPI prescribing. Studies were included if they were conducted in a Western country and focused on oral PPIs for an adult population. By following the Behaviour Change Wheel, we extracted information regarding PPI prescribing behavior, behavioral determinants and intervention strategies.

**Findings:**

We included 74 papers. Most focused on the determinants knowledge and beliefs about consequences. The latter was consistently related to PPI prescribing. Results for knowledge were mixed. Most interventions used education or enablement (e.g., algorithms, quality check improvements, involvement of pharmacists) as strategies. Enablement consistently improved PPI prescribing, while results for education were mixed.

**Interpretation:**

There is an overemphasis on reflective processes in studies on PPI prescribing. Future research should comprehensively identify behavioral determinants, focusing on reflective and impulsive processes, such that interventions can address the most important determinants.

**Supplementary Information:**

The online version contains supplementary material available at 10.1186/s12875-024-02459-5.

Proton pump inhibitors (PPIs) are one of the most commonly prescribed drugs and are the mainstay of the treatment and prevention of various gastrointestinal diseases. In the Netherlands, for example, an estimated 2.6 million people used a PPI in 2022 [1]. National guidelines describe various indications for the use of PPI including treatment of gastroesophageal reflux disease (GERD), peptic ulcer disease, eradication of Helicobacter pylori (HP) and Zollinger-Ellison syndrome. It is also widely used as prophylaxis in high-risk patients to prevent the occurrence of peptic ulcer disease. In addition, PPIs can be prescribed to patients without alarm symptoms if lifestyle modification, acid binders and H2-receptor antagonists fail to relieve reflux symptoms [2–5].

Although PPIs are generally considered effective, safe and well-tolerated, there is a growing body of evidence showing that long-term PPI use is associated with various serious side effects [6]. The most often reported side effects are pneumonia, Clostridium difficile infection, iron/vitamin B12/magnesium deficiency, bone fractures and kidney disease. Dutch primary care studies showed repeatedly that more than half of patients on long-term PPI use lack a valid indication [7–9]. In addition, 32% of patients who initially started on a short-term PPI treatment do not stop after the advised 3 months [9]. This is likely even an underestimation because of the increasing over-the-counter use of PPIs [10]. PPI overprescribing has extensively been confirmed in the international literature [11–14]. In the light of the reported side effects, inappropriate PPI prescribing needs to be addressed [15].

## PPI prescribing behavior

Changing inappropriate prescribing requires changing healthcare professional behavior. There are several reasons for why this may be challenging. First, different specific behaviors occurring in different situations may contribute to PPI overprescribing. Physicians may, for example, decide to initiate PPI prescription for first-time indications, but they may also automatically renew prescriptions during daily routine practice. In these situations, different factors may be of importance in steering prescription behavior. Second, according to Dutch guidelines, the recommended first step in the treatment of stomach related complaints is lifestyle advice. In addition, guidelines advise to taper PPIs in patients with a short-term indication for PPI therapy after 3 months. Both of these behaviors require time investment and patience. Third, some of these behaviors may be ingrained in daily routine practice and be executed habitually. For prescribing physicians, changing PPI overprescribing may require disrupting old habits (e.g., no longer habitually renewing prescriptions). Literature from the behavioral sciences confirms that healthcare professional behavior, and in particular prescribing behavior, is often driven by habits and routines [16, 17]. In these cases, interventions aimed at increasing knowledge or motivation to change will be of limited use and other types of interventions, addressing more automatic influences on behavior, are required. However, current interventions tend to focus almost exclusively on reflective processes like intentions [18], possibly due to lack of knowledge about the role of habits and other automatic influences. Considering these complexities of changing PPI overprescribing behavior, designing successful interventions requires a systematic approach. An important first step is to obtain a more comprehensive picture of the behavioral determinants of PPI prescription. After that, evidence-based behavior change strategies can be selected that are known to target the most important and changeable determinants underlying this behavior.

## Behaviour change wheel

One such evidence-based approach to changing healthcare professional behavior that is increasingly used in designing interventions is the Behaviour Change Wheel [19]. This is a systematic approach to develop interventions, based on three main stages: 1) understanding the behavior, 2) identifying intervention options, and 3) identifying content of the intervention (i.e., Behavior Change Techniques) and implementation options. According to the Behaviour Change Wheel, it is pivotal to spend time and effort on understanding the so-called behavioral determinants. These define the underlying reasons for why people do or do not engage in the desired behavior, and provide starting points for designing interventions that address these relevant determinants. When behavioral determinants are overlooked, interventions will be based on assumptions about the root causes of the target behavior instead of empirical evidence on important determinants. This could potentially create a mismatch between intervention content and relevant determinants.

## The current review

Despite the urgency of addressing PPI overprescription, interventions to limit PPI overprescribing seem to have limited success [20]. To understand how intervention development for PPI overprescribing can be improved, the present scoping review aims to systematically scope the literature through a behavioral lens. Specifically, considering the complexity of overprescribing, a comprehensive picture of what behavioral determinants have been studied and found to be important is required. In particular, given the focus on reflective processes in clinical practice [18], it is relevant to investigate whether indeed the full spectrum of potentially relevant determinants has been studied in scientific literature. In addition, it is unclear what strategies are currently used in interventions targeting PPI overprescribing and whether these actually address the most important determinants.

This scoping review therefore has three main purposes. Following the steps in the Behaviour Change Wheel, first, we aim to reveal what determinants are currently focused on when studying reasons for PPI (de)prescribing. Second, we aim to provide an overview of the strategies current interventions use in their attempts to change PPI (de)prescribing. Finally, we aim to bring these insights together to describe the match between the determinants that have been studied and found to be important with the content of the interventions.

## Method

### Search strategy and selection criteria

Following recent advices [21], we report the results according to the PRISMA-ScR (Preferred Reporting Items for Systematic reviews and Meta-Analyses extension for Scoping Reviews) recommendations [22]. The protocol for this study was registered at the Open Science Framework: https://osf.io/v4axh.

### Literature search

The search strategy was developed in collaboration with a specialized librarian (JS). We sought through the following databases: PubMed, MEDLINE (OVID), Embase (OVID), Web of Science, Cochrane Library, Emcare (OVID), Academic Search Premier (EbscoHOST), and Google Scholar. Contrary to the protocol, we did not search for grey literature with Google and WorldCAT, as an initial attempt to do so did not result in finding relevant documents.

The search strategy included keywords related to prescription behavior (e.g., “prescribing behavior” or “deprescription”), physicians (e.g., “General Practitioners” or "gastroenterologist"), and PPIs (e.g., “Proton Pump Inhibitors” or “omeprazole”). The final and complete search strategy is included in Supplementary Material 1.

### Inclusion criteria

We predefined our inclusion criteria to be the following:

- All studies that evaluate determinants of physicians prescribing behavior of PPIs
- All qualitative and quantitative study designs
- All published articles with available full-texts until the search date

These inclusion criteria were supplemented with additional criteria after screening the first articles:

- Study location in a Western country, as defined by countries in North America, Europe, and Oceania (This focus was chosen due to differences in healthcare systems and because more is known about overprescribing in Western countries) [23]
- Focus on PPI prescribing for an adult patient population
- Focus on oral PPI: All different PPIs will be included (Omeprazole, Esomeprazole, Pantoprazole, Lansoprazole, Rabeprazole)

### Exclusion criteria

- Published in other languages than English or Dutch
- Systematic reviews or studies only describing a study protocol

### Study selection

The search was conducted on 30-06-2022. Title and abstract screening was performed by two reviewers (LG, RMB) independently and systematically according to the above mentioned in- and exclusion criteria. This was done within ASReview (v1.0) by using machine learning technology [24]. Based on previous research showing that 95% of relevant papers can be identified by screening 8% to 33% of all papers [24], we predefined our stop rule for each individual reviewer at a minimum of 33% of all papers and achieving 25 consecutive non-relevant papers. Inconsistencies due to a different decision or because one of the two reviewers had not seen the abstract in ASReview because the algorithm had not selected this paper for screening were discussed by the two reviewers. If necessary, a third reviewer was consulted (JKJ). Simultaneously, a similar screening procedure was conducted for meeting abstracts. For those meeting abstracts that met the inclusion criteria, full-texts were searched. Full-text screening was subsequently done by three reviewers (LG, GB, SK). The remaining meeting abstracts for which no full-texts were available were coded based on all available information in the abstracts. These data can be found in the supplementary online materials.

### Data extraction

Data extraction was performed by four reviewers (RMB, SK, GB, LG) according to a predefined data extraction sheet. Extracted information included amongst others basic study characteristics (e.g., country, design), aims (primary and secondary), methodology (e.g., study population, inclusion criteria, sample size, statistical analysis.), behavioral determinants or intervention strategies (for details see below), outcome (e.g., outcome, method and time of measurement), results (key findings), and conclusions (e.g., conclusion, comments). Data were extracted by at least one reviewer and verified by a second reviewer. The complete data extraction sheet can be found in the supplementary online materials.

#### Behavioral determinants and intervention strategies

For coding the behavioral determinants and intervention strategies, we relied on the Behaviour Change Wheel methodology [19]. Understanding the behavior (Stage 1) starts with defining and specifying the target behavior and the target population by stating who needs to do what, where and when. This is then followed by identifying the determinants that need to be addressed in order to affect the target behavior. We broadly scoped a variety of behaviors relevant to PPI overprescribing, such as first-time indications, renewal of prescriptions, as well as efforts to deprescribe or discontinue prescriptions. Across all these studies, we thus first mapped what the target behavior exactly was.

For studies focused on *understanding* PPI prescription behavior, we coded behavioral determinants according to two often-used frameworks: the COM-B model [19] and the associated, but more refined, Theoretical Domains Framework (TDF) [25]. The COM-B model consists of three overarching predictors of behavior, namely capability, opportunity, and motivation. Capability represents whether someone is able to perform the target behavior, motivation represents whether someone is willing to perform this behavior, and opportunity refers to whether someone’s environment allows one to perform the target behavior. The TDF is a more refined framework based on synthesizing core theoretical constructs from 83 different psychological theories. It consists of fourteen different behavioral domains (e.g., knowledge, skills, beliefs about capabilities and beliefs about consequences) related to either capability, opportunity or motivation.

For intervention studies focused on *changing* PPI prescription behavior, we focused on stage 2 of the Behaviour Change Wheel. We coded intervention functions (i.e., the means by which intervention can change behavior) and policy functions (i.e., policies that support the delivery of the intervention functions). The Behaviour Change Wheel distinguishes between nine intervention functions (e.g., education, persuasion, or training) and seven policy functions (e.g., communication, guidelines, or legislation).

## Results

**Fig. 1:**
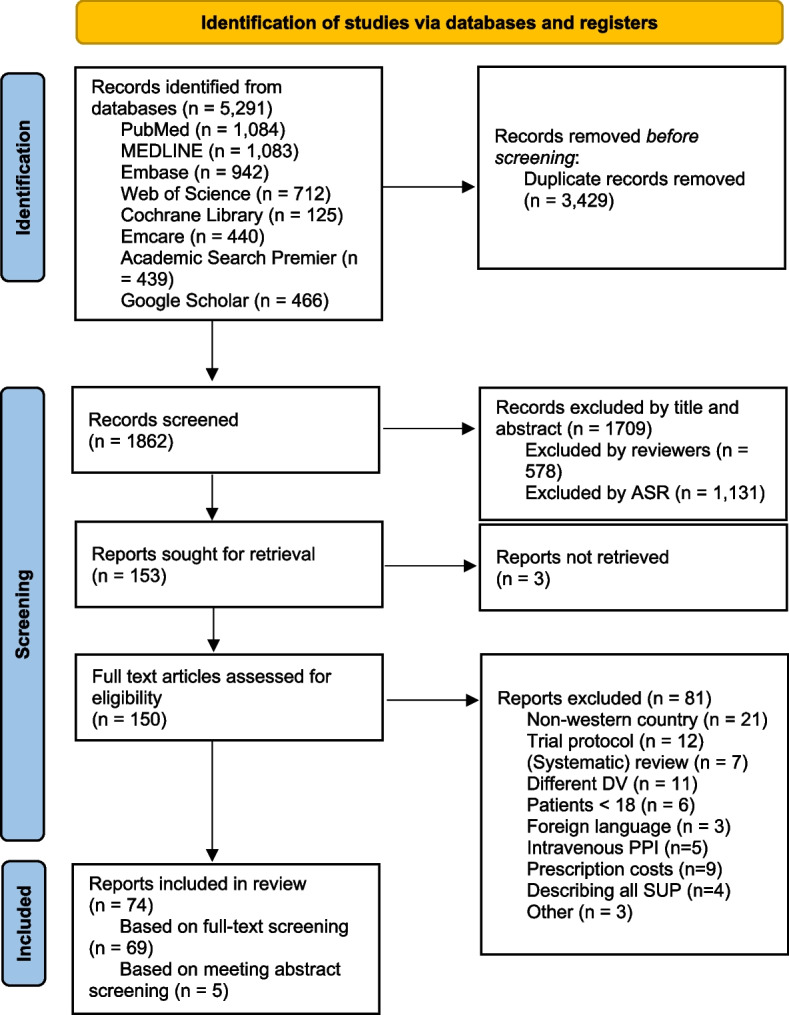
PRISMA flowchart [26]

The literature search identified 5291 articles, which included 3429 duplicates (See Fig. 1). The remaining 1862 articles were screened based on title and abstract, which resulted in 74 papers eligible for data extraction (69 full-texts + 5 meeting abstracts for which full-texts were available). The majority of these papers (*n* = 49, 66%) focused on changing PPI prescription behavior, while a smaller portion of 19 papers (26%) focused on understanding PPI prescription behavior. 6 papers (8%) focused both on understanding and changing PPI prescription behavior. In total, a large majority of 64 papers (86%) used quantitative research methods (e.g., cross-sectional studies or pre-post intervention studies). 7 papers (9%) used qualitative research methods (e.g., semi-structured interviews or focus groups), while 3 papers (4%) used mixed methods. Table 1 displays the results regarding what prescribing behavior took place when, where, and by whom.

**Table 1:** Results regarding who needs to do what, where and when

	*n (%)*
*Who*	
Physicians	74 (100%)
*What*	
Deprescribing	31 (42%)
Appropriate prescribing	24 (32%)
Hypothetical prescription behavior	16 (22%)
Prescribing alternative PPIs	2 (3%)
Prescribing rate	1 (1%)
*Where*	
Outpatient	37 (50%)
Inpatient	28 (38%)
Out- and inpatient	5 (7%)
Population-based	4 (5%)
*Where (specifically)*	
GP	27 (36%)
Nursing home	7 (9%)
Intensive care	3 (4%)
Veteran care	3 (4%)
Discharge to GP	2 (3%)
Veteran GP	1 (1%)
Elderly care	1 (1%)
Hospital and GP	1 (1%)
Intensive care and other units	1 (1%)
*When*	
Discharge	4 (5%)
Not specified	70 (95%)

*PPIs* Proton Pump Inhibitors, *GP* General practice

## Understanding PPI prescription behavior

Studies aimed at describing and understanding PPI prescription behavior (*n* = 25, 34%) focused on different healthcare professionals, different PPI-related behaviors, and on different settings. For these papers, we coded determinants according to the COM-B model and the TDF as further refinement of the COM-B model. Most papers focused on motivation and capability, while less attention was paid to opportunity. When zooming in using the TDF, the two determinants that were most often focused on are knowledge (*n* = 15, 60%) and beliefs about consequences (*n* = 13, 52%). Other domains were hardly ever addressed (e.g., social influences or beliefs about capabilities) or not at all (e.g., behavioral regulation or goals). For a complete overview, see Table 2.

**Table 2:** Overview of behavioral determinants identified in studies about understanding PPI prescription behavior

COM-B	*n (%)*	TDF	*n (%)*
Psychological capability	15 (60%)	Knowledge	15 (60%)
		Memory, attention, and decision processes	2 (8%)
		Behavioral regulation	-
Physical capability	1 (4%)	Skills	2 (8%)
Reflective motivation	14 (56%)	Social/Professional role and identity	2 (8%)
		Beliefs about capabilities	3 (12%)
		Optimism	-
		Intentions	1 (4%)
		Goals	-
		Beliefs about consequences	13 (52%)
Automatic motivation	4 (16%)	Reinforcement	3 (12%)
		Emotion	1 (4%)
Social opportunity	4 (16%)	Social influences	4 (16%)
Physical opportunity	3 (12%)	Environmental context and resources	3 (12%)

*COM-B* Capability, Opportunity, Motivation, Behavior, *TDF* Theoretical Domains Framework

For the two most often studied domains, we looked into the importance of these determinants. For knowledge, studies reported heterogeneous results. While some studies revealed good levels of knowledge about appropriate prescribing techniques and side effects [27–29], others pointed out room for improvement [30], especially with respect to specific risks of PPIs in patients with rare diseases in tertiary care settings [31]. Moreover, qualitative studies identified (lack of) knowledge as one of the major barriers towards appropriate prescribing or deprescribing [31, 32]. Quantitative work also identified an association between knowledge (of indications or side effects) and prescribing behavior [30].

Regarding beliefs about consequences, these were mostly expressed by majorities of physicians as concerns about adverse effects or events, especially regarding long-term consequences [27, 28, 32]. Such concerns were also associated with lower self-reported PPI prescription rates [30, 33, 34].

Other, more practical, barriers towards appropriate PPI prescribing were fear of legal repercussions [30], time constraints [31], and waiting time for an endoscopy or hospital appointment [35].

## Changing PPI prescription behavior

Intervention studies (*n* = 55, 74%) comprised of longitudinal studies in which a policy change was evaluated (prospectively or retrospectively) as well as studies that evaluated a specific intervention as part of the study itself. The majority of these studies included publication or dissemination of (new) guidelines [36, 37], implementation of algorithms [38, 39], and pharmacist oversight or interventions [40, 41].

In terms of intervention functions, interventions mostly focused on educational purposes (*n* = 29, 53%). Enablement was also often used (*n* = 23, 42%), while a smaller portion of interventions focused on persuasion (*n* = 11, 20%) and environmental restructuring (*n* = 9, 16%).

For the two most often used intervention functions, we looked into the reported effectiveness. For education (e.g., educational leaflets mailed to prescribers or an internet course on medication prescribing), results showed mixed effects. Most interventions focusing on education reported reductions in PPI prescriptions [42–48], but most of these were multifaceted interventions [44, 45, 47, 48]. Other multifaceted interventions including an education component were ineffective [49]. If the intervention purely focused on education, results were also mixed, with some reporting no effect [13], some reporting limited impact (e.g., a small decrease in monthly PPI dispensing, but no effects on switching or discontinuation) [50], and others reporting desired effects [42, 43, 46].

Regarding enablement, the vast majority of studies reported desired effects on (in)appropriate PPI prescribing and deprescribing outcomes [12, 38, 39, 41, 44, 47, 48, 51–62]. These studies included algorithms, other deprescribing tools, quality check interventions or other quality improvement strategies, and often involved pharmacists. Only one study that focused on improving practice for Helicobacter pylori eradication concluded their intervention including enablement was not a worthwhile strategy [63].

Finally, in papers where policy functions could be identified, these mostly included communication/marketing (*n* = 29, 53%) and service provision (*n* = 26, 47%), followed by guidelines (*n* = 18, 33%). For a complete overview of intervention and policy functions, see Table 3.

**Table 3:** Overview of intervention functions and policy functions identified in studies about changing PPI prescription behavior

	n (%)
*Intervention function*	
Education	29 (53%)
Enablement	23 (42%)
Persuasion	11 (20%)
Environmental restructuring	9 (16%)
Restriction	4 (7%)
Incentivization	3 (5%)
Training	2 (4%)
Modeling	1 (2%)
Coercion	-
*Policy function*	
Communication/marketing	29 (53%)
Service provision	26 (47%)
Guidelines	18 (33%)
Environmental/social planning	7 (13%)
Regulation	4 (7%)
Fiscal measures	2 (4%)
Legislation	-

## Discussion

This systematic scoping review aimed first to scope which determinants of physicians’ PPI prescribing behavior have been studied and found to be important, second to map which types of strategies in terms of interventions and policy functions are used in interventions, and third whether these match with the determinants found to be of importance. The review has clearly shown that there is a large emphasis on reflective processes such as knowledge and beliefs about consequences in studies about underlying determinants. While these determinants were studied the most, results regarding the importance of knowledge for affecting PPI prescribing behavior were overall rather mixed, although some studies indeed pointed out that a lack of knowledge can pose a barrier towards appropriate prescribing or successful deprescribing efforts [30–32]. Studies consistently pointed out that concerns about side effects of long-term PPI use are related to lower rates of self-reported PPI prescribing behavior [30, 33, 34], which seems legitimate given increasing knowledge about this topic [6]. The most frequently mentioned concerns for side effects related to bone fractures and osteoporosis [33]. One study conducted in the USA further revealed that fear legal repercussions following a gastrointestinal bleeding without having prescribed PPIs was associated with higher prescribing rates [30].

Intervention studies had a very strong focus on education and enablement. Educational interventions for example included education about guidelines or long-term consequences of PPI use. Results regarding the effectiveness of educational interventions were mixed, with some studies reporting desired effects [42–48], some reporting limited effects [50], and some reporting no effects [13]. However, most of these interventions were multifaceted and included other intervention functions as well. Previous research on changing physician behavior has revealed promise of such multifaceted interventions [64–66], but it is impossible to ascribe intervention success to one component like education if more intervention functions are used. For enablement, very consistent positive effects were observed [12, 38, 39, 41, 44, 47, 48, 51–62]. These effective interventions mostly focused on deprescribing and involved algorithms, quality check improvements and involvement of pharmacists. The finding that interventions mostly included publication of new guidelines, implementation of algorithms, audits and feedback, and pharmacist interventions is largely in line with other reviews about interventions to change healthcare professional (prescribing) behavior [67].

Taken together, this means that the studies investigating behavioral determinants have currently been confined to addressing only a small set of determinants. This spotlight on only a few potential determinants limits the possibility to conclude whether interventions currently target the right determinants. Still, within the interventions scoped, education may be suitable to increase knowledge and beliefs about consequences, the two determinants that have been studied most frequently. Knowledge is an essential building block for changing behavior, but the findings suggest that there may be little to improve on, as most physicians already express high levels of concern regarding potential side effects or adverse events [27, 28, 32]. This may be a potential explanation for the limited effects of educational interventions. Enablement, on the other hand, can be used to increase memory, attention and decision-making processes, environmental context and resources, and social influences. While our scoping review identified little focus on such determinants in studies describing prescription behavior, it also revealed consistent positive effects of enablement on PPI prescription behavior.

### Implications

The most important implication of this scoping review is that there is an overemphasis on reflective processes, both in studies describing PPI prescription behavior and in intervention studies. Determinants like knowledge and beliefs about consequences received most attention and most studies focused on educational purposes. At the same time, surprisingly little is known in the scoped literature about more practical barriers like lack of time and resources. We only found one study that reported that a lack of time can pose a barrier towards discussing deprescribing with patients [31], and one other study that reported on waiting time for a hospital appointment or endoscopy as a practical barrier [35]. Importantly, more automatic determinants like habits and routines have also received considerably less attention thus far. Given increasing attention to such nonreflective processes in research on healthcare professional prescription behavior [16, 17], it is likely that these processes are also relevant for PPI prescription behavior and possible candidates for successful interventions.

Given the confined picture and lack of a comprehensive overview of behavioral determinants studied in the literature, it is important to engage in systematic intervention development such that the strategies that make up an intervention indeed address the relevant target determinants. The behavioral sciences have procedures and tools for this (like the Behaviour Change Wheel [19]), and behavior change interventions focused on changing healthcare professional behavior are likely to benefit from such expertise. Moreover, integrating expertise from the behavioral sciences in interdisciplinary collaborations can aid in studying a wider array of determinants and incorporating different strategies in interventions. To illustrate, if automatic processes are indeed found to be important, different strategies like implementation intentions, the use of prompts, nudging or removal or contextual cues may be needed [68].

### Strengths and limitations

To the best of our knowledge, this scoping review is the first review to outline determinants of PPI prescription behavior and interventions to increase appropriate prescribing. Given high rates of PPI use in Western countries [11–14], high rates of inappropriate prescriptions [7–9], and concerns about long-term use [6], inappropriate prescribing urgently needs to be addressed. The current scoping review incorporates behavioral expertise to provide a comprehensive overview of relevant determinants and intervention strategies. The broad scope of this review allowed us to fully map current evidence regarding PPI prescription behavior in all its diversity.

Despite the relevance of this review, there are important limitations that should be considered. First, the quality of this review is limited by a lack of precision in reporting of target behaviors. In order to fully understand behavioral problems, it is essential to first specify it in detail. Yet, many of the reported studies do not provide adequate information in terms of *who* needs to do *what, where,* and, *when*. For example, studies generally made use of data that did not distinguish between first-time indications or renewal of prescription by the physician, and it was often unclear whether patients had a short-term indication or whether they were long-term users. As concerns have been raised about side effects of long-term PPI use [6], such information about the target behavior is relevant to design interventions that target inappropriate or long-term prescribing specifically, rather than appropriate or shorter-term usage. Moreover, almost none of the studies were specific about when PPIs were prescribed (e.g., at discharge). An inadequate specification of the behavior under study hinders systematic evidence accumulation [69].

Second, given a lack of precision in reporting of interventions, we were only able to apply stage 1 and 2 of the Behaviour Change Wheel to the literature on PPI overprescribing. Current consensus in the behavioral sciences is that interventions should describe what Behavior Change Techniques are used (Stage 3). It was, however, impossible to code on this more specific level given inaccurate descriptions of most interventions. To illustrate, many studies report on implementation of guidelines. These are likely to have an educational function and target knowledge of physicians, but this is not always explicitly described or assessed. Besides, it was often unclear what the focus of the educational efforts were and how exactly education was delivered to the physicians. As such, interventions that have been rolled out are also not described in sufficient detail to evaluate whether they are indeed targeting the relevant determinants. Future research should therefore more systematically describe what Behavior Change Techniques the intervention was comprised of to achieve better science accumulation.

Finally, as the review focused on identifying behavioral determinants of PPI prescribing, we were not able to incorporate insights about systemic influences, which are generally known to impact (de)prescribing. To illustrate, deprescribing interventions are influenced by organizational, interprofessional, and patient-specific barriers, which can be exacerbated by involvement from multiple prescribers in a fragmented healthcare system [70]. Inadequate communication among patients, prescribers and pharmacies may lead to a lack of medication oversight, with no single provider taking responsibility for prolonged medication use. This communication gap can result in continued prescriptions for medication such as PPI, diminishing the efficacy of deprescribing interventions. Such aspects of continuity of care (COC) [71] were not identified in the current review, while recent studies indicate that COC might be an important factor in medication management [72]. Lack of COC is associated with less changes in patient medication and less deprescribing [73]. Empanelment systems [74], on the other hand, have been suggested to improve COC, ensuring a systematic approach to patient care that enables healthcare providers to identify and manage specific patient groups and improve their medication monitoring. This could thereby facilitate deprescribing [75]. Altogether, this shows that both the behavioral and the medical sciences suggest important additional reasons for (in)appropriate prescribing. Therefore, we encourage scholars to incorporate interpersonal and organizational factors that can impact PPI prescribing in future research.

## Conclusion

This scoping review revealed that there is an overemphasis on reflective processes both in studies describing PPI prescription behavior (knowledge and beliefs about consequences) and studies intervening on PPI prescription behavior (education). Enabling physicians to change their prescription behavior by means of providing algorithms or pharmacist support was found to be most effective. Future research should comprehensively aim to identify behavioral determinants, focusing both on reflective and impulsive processes, and the full spectrum of determinants that have been uniquely identified within the TDF. Similarly, interventions should be developed systematically rather than being based on assumptions or limited knowledge of underlying reasons for the various types of PPI-related behaviors. A comprehensive picture of relevant determinants will allow for addressing the root causes of the behavioral problem, by developing an intervention that makes use of the right strategies that target these causes.

### Supplementary Information

Supplementary Material 1. 

Supplementary Material 2.

Supplementary Material 3. 

Supplementary Material 4. 

## Data Availability

The search strategy and data extraction sheet are included as Supplementary Materials to this article. All relevant information is also available on the Open Science Framework: https://osf.io/6ysvc/.
