# Supplementary material for: Determinants of and interventions for Proton Pump Inhibitor prescription behavior: A systematic scoping review
Source: BMC Prim Care. 2024 Jun 11;25:208. doi: 10.1186/s12875-024-02459-5 (PMC11165893; doi:10.1186/s12875-024-02459-5)
Supplement: Supplementary file 1 — Supplementary Material 1. [file 12875_2024_2459_MOESM1_ESM.docx]

**Supplementary file 1**

Search strategies:

1. PubMed

(("prescribing behavior"[tw] OR "prescribing behaviors"[tw] OR "prescribing behaviour"[tw] OR "prescribing behaviours"[tw] OR "prescription behavior"[tw] OR "prescription behaviors"[tw] OR "prescription behaviour"[tw] OR "prescription behaviours"[tw] OR "Prescribing Pattern"[tw] OR "Prescribing Patterns"[tw] OR "Prescription Pattern"[tw] OR "Prescription Patterns"[tw] OR "Prescribing Adherence"[tw] OR "Prescription Adherence"[tw] OR "Prescription Non Adherence"[tw] OR "Prescription Nonadherence"[tw] OR "Prescribing Compliance"[tw] OR "Prescription Compliance"[tw] OR "Prescription Non Compliance"[tw] OR "Prescription Noncompliance"[tw] OR "prescribing practice"[tw] OR "prescribing practices"[tw] OR "prescription practice"[tw] OR "prescription practices"[tw] OR "Deprescriptions"[mesh] OR "Drug Tapering"[mesh] OR "Inappropriate Prescribing"[mesh] OR "Medication Errors"[mesh] OR "Drug Utilization"[mesh] OR "Drug Prescriptions"[mesh] OR "tapering"[tw] OR "taper*"[tw] OR "deprescribing"[tw] OR "deprescrib*"[tw] OR "deprescription"[tw] OR "deprescriptions"[tw] OR "deprescrip*"[tw] OR (("Drug Utilization"[mesh] OR "Drug Prescriptions"[mesh] OR "prescription"[tw] OR "prescriptions"[tw] OR "prescrip*"[tw] OR "prescribing"[tw] OR "prescribe"[tw] OR "prescrib*"[tw] OR "medication"[tw] OR "medications"[tw] OR "medicat*"[tw]) AND ("appropriate"[tw] OR "inappropriate"[tw] OR "appropriat*"[tw] OR "inappropriat*"[tw] OR "cessation"[tw] OR "tapering"[tw] OR "taper*"[tw] OR "stopping"[tw] OR "stop"[tw] OR "deprescribing"[tw] OR "deprescrib*"[tw] OR "deprescription"[tw] OR "deprescriptions"[tw] OR "deprescrip*"[tw] OR "implementation"[tw] OR "implement*"[tw] OR "deimplementation"[tw] OR "deimplement*"[tw])) OR "deprescrib*"[tiab] OR "deprescriptions"[mesh] OR (("medication*"[tiab] OR "prescribing"[tiab]) AND "inappropriate"[tiab]) OR "polypharmacy"[tiab] OR "discontinu*"[tiab] OR ("withdraw*"[tiab] AND "medication*"[tiab]) OR (("medication*"[tiab] OR "drugs"[tiab] OR "prescribing"[tiab] OR "inappropriate"[tiab]) AND "reduc*"[tiab]) OR "inappropriate prescribing"[mesh] OR ("review*"[tiab] AND "medication"[tiab]) OR ("dose reduction"[tiab] OR "taper*"[tiab]) OR "appropriate"[tw] OR "appropriat*"[tw] OR "inappropriate"[tw] OR "inappropriat*"[tw]) AND ("General Practitioners"[Mesh] OR "General Practitioner"[tw] OR "General Practitioners"[tw] OR "General Practice physician"[tw] OR "General Practice physicians"[tw] OR "General Practice"[Mesh] OR "General Practice"[tw] OR "Physicians, Family"[mesh] OR "Family Physician"[tw] OR "Family Physicians"[tw] OR "Family Practice"[Mesh] OR "Family Practice"[tw] OR "Physicians, Primary Care"[mesh] OR "Primary Care Physician"[tw] OR "Primary Care Physicians"[tw] OR "Primary Health Care"[Mesh:NoExp] OR "Primary Care"[tw] OR "Primary Health Care"[tw] OR "Primary Healthcare"[tw] OR "Health Personnel"[Mesh:NoExp] OR "Health Care Providers"[tw] OR "Health Care Provider"[tw] OR "Healthcare Providers"[tw] OR "Healthcare Provider"[tw] OR "Healthcare Workers"[tw] OR "Healthcare Worker"[tw] OR "Health Care Professionals"[tw] OR "Health Care Professional"[tw] OR "Healthcare Professionals"[tw] OR "Healthcare Professional"[tw] OR "Physicians"[Mesh] OR "Physicians"[tw] OR "Physician"[tw] OR "Physician*"[tw] OR "practitioner"[tw] OR "practitioners"[tw] OR "doctor"[tw] OR "doctors"[tw] OR "doctor*"[tw] OR "gastroenterologist"[tw] OR "gastroenterologists"[tw] OR "gastroenterologist*"[tw] OR "internist"[tw] OR "internists"[tw] OR "internist*"[tw] OR "geriatrician"[tw] OR "geriatricians"[tw] OR "nursing home physician"[tw] OR "nursing home physicians"[tw] OR "nursing home specialist"[tw] OR "nursing home specialists"[tw] OR "Pediatricians"[Mesh] OR "pediatrician"[tw] OR "pediatricians"[tw] OR "paediatrician"[tw] OR "paediatricians"[tw] OR "resident"[tw] OR "residents"[tw] OR "Internship and Residency"[Mesh] OR "Pharmacists"[Mesh] OR "pharmacist"[tw] OR "pharmacists"[tw]) AND ("Proton Pump Inhibitors"[Mesh] OR "Proton Pumps/antagonists and inhibitors"[Mesh] OR "Proton Pump Inhibitors"[Pharmacological Action] OR "Proton Pump Inhibitors"[tw] OR "Proton Pump Inhibitor"[tw] OR (("PPI"[tw] OR "PPIs"[tw]) AND ("dyspepsia"[tw] OR "dyspeptic"[tw] OR "reflux"[tw] OR "gastric"[tw] OR "stomach"[tw])) OR "1-(2-methyl-4-methoxyphenyl)-4-((2-hydroxyethyl)amino)-6-trifluoromethoxy-2,3-dihydropyrrolo(3,2-c)quinoline"[Supplementary Concept] OR "1-(2-methylphenyl)-4-methylamino-6-methyl-2,3-dihydropyrrolo(3,2-c)quinoline"[Supplementary Concept] OR "2,3-dimethyl-8-(2-ethyl-6-methylbenzylamino)imidazo(1,2-a)pyridine-6-carboxamide"[Supplementary Concept] OR "2-(2-ethylaminobenzylsulfinyl)-5,6-dimethoxybenzimidazole"[Supplementary Concept] OR "3-(3-(ethoxycarbonyl)propionyl)-8-methoxy-4-((2-methylphenyl)amino)quinoline"[Supplementary Concept] OR "3-butyryl-4-(2-methylphenylamino)-8-(2-hydroxyethoxy)quinoline"[Supplementary Concept] OR "3-butyryl-4-(5R-methylbenzylamino)-8-ethoxy-1,7-naphthyridine"[Supplementary Concept] OR "8-((2-benzimidazolyl)sulfinylmethyl)-1-ethyl-1,2,3,4-tetrahydroquinoline"[Supplementary Concept] OR "8-(2-ethyl-6-methylbenzylamino)-3-hydroxymethyl-2-methylimidazo(1,2-a)pyridine-6-carboxamide"[Supplementary Concept] OR "B 823-10"[Supplementary Concept] OR "BY 831-78"[Supplementary Concept] OR "BY 841"[Supplementary Concept] OR "cassigarol A"[Supplementary Concept] OR "Dexlansoprazole"[mesh] OR "Esomeprazole"[mesh] OR "ethyl 2-((1H-benzimidazol-2-yl)sulfinylmethyl)-4-dimethylamino-5-pyrimidinecarboxylate"[Supplementary Concept] OR "Lansoprazole"[mesh] OR "Omeprazole"[mesh] OR "omeprazole, sodium bicarbonate drug combination"[Supplementary Concept] OR "Pantoprazole"[mesh] OR "Rabeprazole"[mesh] OR "S 1924"[Supplementary Concept] OR "salvianolic acid A"[Supplementary Concept] OR "scopadulciol"[Supplementary Concept] OR "SK^ and F 96079"[Supplementary Concept] OR "SPI 447"[Supplementary Concept] OR "T 330"[Supplementary Concept] OR "timoprazole"[Supplementary Concept] OR "TY 11345"[Supplementary Concept] OR "xanthoangelol"[Supplementary Concept] OR "YJA 20379-1"[Supplementary Concept] OR "YJA 20379-5"[Supplementary Concept] OR "YJA 20379-6"[Supplementary Concept] OR "1-(2-methyl-4-methoxyphenyl)-4-((2-hydroxyethyl)amino)-6-trifluoromethoxy-2,3-dihydropyrrolo(3,2-c)quinoline"[tw] OR "1-(2-methylphenyl)-4-methylamino-6-methyl-2,3-dihydropyrrolo(3,2-c)quinoline"[tw] OR "2,3-dimethyl-8-(2-ethyl-6-methylbenzylamino)imidazo(1,2-a)pyridine-6-carboxamide"[tw] OR "2-(2-ethylaminobenzylsulfinyl)-5,6-dimethoxybenzimidazole"[tw] OR "3-(3-(ethoxycarbonyl)propionyl)-8-methoxy-4-((2-methylphenyl)amino)quinoline"[tw] OR "3-butyryl-4-(2-methylphenylamino)-8-(2-hydroxyethoxy)quinoline"[tw] OR "3-butyryl-4-(5R-methylbenzylamino)-8-ethoxy-1,7-naphthyridine"[tw] OR "8-((2-benzimidazolyl)sulfinylmethyl)-1-ethyl-1,2,3,4-tetrahydroquinoline"[tw] OR "8-(2-ethyl-6-methylbenzylamino)-3-hydroxymethyl-2-methylimidazo(1,2-a)pyridine-6-carboxamide"[tw] OR "B 823-10"[tw] OR "BY 831-78"[tw] OR "BY 841"[tw] OR "cassigarol A"[tw] OR "Dexlansoprazole"[tw] OR "Esomeprazole"[tw] OR "ethyl 2-((1H-benzimidazol-2-yl)sulfinylmethyl)-4-dimethylamino-5-pyrimidinecarboxylate"[tw] OR "Lansoprazole"[tw] OR "Omeprazole"[tw] OR "omeprazole, sodium bicarbonate drug combination"[tw] OR "Pantoprazole"[tw] OR "Rabeprazole"[tw] OR "S 1924"[tw] OR "salvianolic acid A"[tw] OR "scopadulciol"[tw] OR "SK^ and F 96079"[tw] OR "SPI 447"[tw] OR "T 330"[tw] OR "timoprazole"[tw] OR "TY 11345"[tw] OR "xanthoangelol"[tw] OR "YJA 20379-1"[tw] OR "YJA 20379-5"[tw] OR "YJA 20379-6"[tw]) AND (english[la] OR dutch[la]))

2. MEDLINE (OVID)

(("prescribing behavior".mp OR "prescribing behaviors".mp OR "prescribing behaviour".mp OR "prescribing behaviours".mp OR "prescription behavior".mp OR "prescription behaviors".mp OR "prescription behaviour".mp OR "prescription behaviours".mp OR "Prescribing Pattern".mp OR "Prescribing Patterns".mp OR "Prescription Pattern".mp OR "Prescription Patterns".mp OR "Prescribing Adherence".mp OR "Prescription Adherence".mp OR "Prescription Non Adherence".mp OR "Prescription Nonadherence".mp OR "Prescribing Compliance".mp OR "Prescription Compliance".mp OR "Prescription Non Compliance".mp OR "Prescription Noncompliance".mp OR "prescribing practice".mp OR "prescribing practices".mp OR "prescription practice".mp OR "prescription practices".mp OR exp "Deprescriptions"/ OR exp "Drug Tapering"/ OR exp "Inappropriate Prescribing"/ OR exp "Medication Errors"/ OR exp "Drug Utilization"/ OR exp "Drug Prescriptions"/ OR "tapering".mp OR "taper*".mp OR "deprescribing".mp OR "deprescrib*".mp OR "deprescription".mp OR "deprescriptions".mp OR "deprescrip*".mp OR ((exp "Drug Utilization"/ OR exp "Drug Prescriptions"/ OR "prescription".mp OR "prescriptions".mp OR "prescrip*".mp OR "prescribing".mp OR "prescribe".mp OR "prescrib*".mp OR "medication".mp OR "medications".mp OR "medicat*".mp) AND ("appropriate".mp OR "inappropriate".mp OR "appropriat*".mp OR "inappropriat*".mp OR "cessation".mp OR "tapering".mp OR "taper*".mp OR "stopping".mp OR "stop".mp OR "deprescribing".mp OR "deprescrib*".mp OR "deprescription".mp OR "deprescriptions".mp OR "deprescrip*".mp OR "implementation".mp OR "implement*".mp OR "deimplementation".mp OR "deimplement*".mp)) OR "deprescrib*".ti,ab OR exp "deprescriptions"/ OR (("medication*".ti,ab OR "prescribing".ti,ab) AND "inappropriate".ti,ab) OR "polypharmacy".ti,ab OR "discontinu*".ti,ab OR ("withdraw*".ti,ab AND "medication*".ti,ab) OR (("medication*".ti,ab OR "drugs".ti,ab OR "prescribing".ti,ab OR "inappropriate".ti,ab) AND "reduc*".ti,ab) OR exp "inappropriate prescribing"/ OR ("review*".ti,ab AND "medication".ti,ab) OR ("dose reduction".ti,ab OR "taper*".ti,ab) OR "appropriate".mp OR "appropriat*".mp OR "inappropriate".mp OR "inappropriat*".mp) AND (exp "General Practitioners"/ OR "General Practitioner".mp OR "General Practitioners".mp OR "General Practice physician".mp OR "General Practice physicians".mp OR exp "General Practice"/ OR "General Practice".mp OR exp "Physicians, Family"/ OR "Family Physician".mp OR "Family Physicians".mp OR exp "Family Practice"/ OR "Family Practice".mp OR exp "Physicians, Primary Care"/ OR "Primary Care Physician".mp OR "Primary Care Physicians".mp OR "Primary Health Care"/ OR "Primary Care".mp OR "Primary Health Care".mp OR "Primary Healthcare".mp OR "Health Personnel"/ OR "Health Care Providers".mp OR "Health Care Provider".mp OR "Healthcare Providers".mp OR "Healthcare Provider".mp OR "Healthcare Workers".mp OR "Healthcare Worker".mp OR "Health Care Professionals".mp OR "Health Care Professional".mp OR "Healthcare Professionals".mp OR "Healthcare Professional".mp OR exp "Physicians"/ OR "Physicians".mp OR "Physician".mp OR "Physician*".mp OR "practitioner".mp OR "practitioners".mp OR "doctor".mp OR "doctors".mp OR "doctor*".mp OR "gastroenterologist".mp OR "gastroenterologists".mp OR "gastroenterologist*".mp OR "internist".mp OR "internists".mp OR "internist*".mp OR "geriatrician".mp OR "geriatricians".mp OR "nursing home physician".mp OR "nursing home physicians".mp OR "nursing home specialist".mp OR "nursing home specialists".mp OR exp "Pediatricians"/ OR "pediatrician".mp OR "pediatricians".mp OR "paediatrician".mp OR "paediatricians".mp OR "resident".mp OR "residents".mp OR exp "Internship and Residency"/ OR exp "Pharmacists"/ OR "pharmacist".mp OR "pharmacists".mp) AND (exp "Proton Pump Inhibitors"/ OR exp "Proton Pumps"/ai OR "Proton Pump Inhibitors".mp OR "Proton Pump Inhibitor".mp OR (("PPI".mp OR "PPIs".mp) AND ("dyspepsia".mp OR "dyspeptic".mp OR "reflux".mp OR "gastric".mp OR "stomach".mp)) OR "1-(2-methyl-4-methoxyphenyl)-4-((2-hydroxyethyl)amino)-6-trifluoromethoxy-2,3-dihydropyrrolo(3,2-c)quinoline"/ OR "1-(2-methylphenyl)-4-methylamino-6-methyl-2,3-dihydropyrrolo(3,2-c)quinoline"/ OR "2,3-dimethyl-8-(2-ethyl-6-methylbenzylamino)imidazo(1,2-a)pyridine-6-carboxamide"/ OR "2-(2-ethylaminobenzylsulfinyl)-5,6-dimethoxybenzimidazole"/ OR "3-(3-(ethoxycarbonyl)propionyl)-8-methoxy-4-((2-methylphenyl)amino)quinoline"/ OR "3-butyryl-4-(2-methylphenylamino)-8-(2-hydroxyethoxy)quinoline"/ OR "3-butyryl-4-(5R-methylbenzylamino)-8-ethoxy-1,7-naphthyridine"/ OR "8-((2-benzimidazolyl)sulfinylmethyl)-1-ethyl-1,2,3,4-tetrahydroquinoline"/ OR "8-(2-ethyl-6-methylbenzylamino)-3-hydroxymethyl-2-methylimidazo(1,2-a)pyridine-6-carboxamide"/ OR "B 823-10"/ OR "BY 831-78"/ OR "BY 841"/ OR "cassigarol A"/ OR "Dexlansoprazole"/ OR "Esomeprazole"/ OR "ethyl 2-((1H-benzimidazol-2-yl)sulfinylmethyl)-4-dimethylamino-5-pyrimidinecarboxylate"/ OR "Lansoprazole"/ OR "Omeprazole"/ OR "omeprazole, sodium bicarbonate drug combination"/ OR "Pantoprazole"/ OR "Rabeprazole"/ OR "S 1924"/ OR "salvianolic acid A"/ OR "scopadulciol"/ OR "SK^ and F 96079"/ OR "SPI 447"/ OR "T 330"/ OR "timoprazole"/ OR "TY 11345"/ OR "xanthoangelol"/ OR "YJA 20379-1"/ OR "YJA 20379-5"/ OR "YJA 20379-6"/ OR "1-(2-methyl-4-methoxyphenyl)-4-((2-hydroxyethyl)amino)-6-trifluoromethoxy-2,3-dihydropyrrolo(3,2-c)quinoline".mp OR "1-(2-methylphenyl)-4-methylamino-6-methyl-2,3-dihydropyrrolo(3,2-c)quinoline".mp OR "2,3-dimethyl-8-(2-ethyl-6-methylbenzylamino)imidazo(1,2-a)pyridine-6-carboxamide".mp OR "2-(2-ethylaminobenzylsulfinyl)-5,6-dimethoxybenzimidazole".mp OR "3-(3-(ethoxycarbonyl)propionyl)-8-methoxy-4-((2-methylphenyl)amino)quinoline".mp OR "3-butyryl-4-(2-methylphenylamino)-8-(2-hydroxyethoxy)quinoline".mp OR "3-butyryl-4-(5R-methylbenzylamino)-8-ethoxy-1,7-naphthyridine".mp OR "8-((2-benzimidazolyl)sulfinylmethyl)-1-ethyl-1,2,3,4-tetrahydroquinoline".mp OR "8-(2-ethyl-6-methylbenzylamino)-3-hydroxymethyl-2-methylimidazo(1,2-a)pyridine-6-carboxamide".mp OR "B 823-10".mp OR "BY 831-78".mp OR "BY 841".mp OR "cassigarol A".mp OR "Dexlansoprazole".mp OR "Esomeprazole".mp OR "ethyl 2-((1H-benzimidazol-2-yl)sulfinylmethyl)-4-dimethylamino-5-pyrimidinecarboxylate".mp OR "Lansoprazole".mp OR "Omeprazole".mp OR "omeprazole, sodium bicarbonate drug combination".mp OR "Pantoprazole".mp OR "Rabeprazole".mp OR "S 1924".mp OR "salvianolic acid A".mp OR "scopadulciol".mp OR "SK^ and F 96079".mp OR "SPI 447".mp OR "T 330".mp OR "timoprazole".mp OR "TY 11345".mp OR "xanthoangelol".mp OR "YJA 20379-1".mp OR "YJA 20379-5".mp OR "YJA 20379-6".mp) AND (english.la OR dutch.la))

3. Embase (OVID)

(("prescribing behavior".ti,ab OR "prescribing behaviors".ti,ab OR "prescribing behaviour".ti,ab OR "prescribing behaviours".ti,ab OR "prescription behavior".ti,ab OR "prescription behaviors".ti,ab OR "prescription behaviour".ti,ab OR "prescription behaviours".ti,ab OR "Prescribing Pattern".ti,ab OR "Prescribing Patterns".ti,ab OR "Prescription Pattern".ti,ab OR "Prescription Patterns".ti,ab OR "Prescribing Adherence".ti,ab OR "Prescription Adherence".ti,ab OR "Prescription Non Adherence".ti,ab OR "Prescription Nonadherence".ti,ab OR "Prescribing Compliance".ti,ab OR "Prescription Compliance".ti,ab OR "Prescription Non Compliance".ti,ab OR "Prescription Noncompliance".ti,ab OR exp *"prescribing practice"/ OR "prescribing practice".ti,ab OR "prescribing practices".ti,ab OR "prescription practice".ti,ab OR "prescription practices".ti,ab OR exp *"Deprescription"/ OR exp "Drug Dose Reduction"/ OR exp *"Inappropriate Prescribing"/ OR exp *"Medication Error"/ OR exp *"Drug Utilization"/ OR exp *"Prescriptions"/ OR "tapering".ti,ab OR "taper*".ti,ab OR "deprescribing".ti,ab OR "deprescrib*".ti,ab OR "deprescription".ti,ab OR "deprescriptions".ti,ab OR "deprescrip*".ti,ab OR ((exp *"Drug Utilization"/ OR exp *"Prescriptions"/ OR "prescription".ti,ab OR "prescriptions".ti,ab OR "prescrip*".ti,ab OR "prescribing".ti,ab OR "prescribe".ti,ab OR "prescrib*".ti,ab OR "medication".ti,ab OR "medications".ti,ab OR "medicat*".ti,ab) AND ("appropriate".ti,ab OR "inappropriate".ti,ab OR "appropriat*".ti,ab OR "inappropriat*".ti,ab OR "cessation".ti,ab OR "tapering".ti,ab OR "taper*".ti,ab OR "stopping".ti,ab OR "stop".ti,ab OR "deprescribing".ti,ab OR "deprescrib*".ti,ab OR "deprescription".ti,ab OR "deprescriptions".ti,ab OR "deprescrip*".ti,ab OR "implementation".ti,ab OR "implement*".ti,ab OR "deimplementation".ti,ab OR "deimplement*".ti,ab)) OR "deprescrib*".ti,ab OR ((review* ADJ3 medication*).ti,ab) OR (((medication* OR medicines OR prescribing) ADJ4 inappropriate).ti,ab) OR "potentially inappropriate".ti,ab OR ((reduc* ADJ5 medication*).ti,ab) OR "polypharmacy".ti,ab OR "discontinu*".ti,ab OR "withdraw*".ti,ab OR ((reducing ADJ1 (drug* OR inappropriate OR frid)).ti,ab) OR exp "polypharmacy"/ OR exp "medication therapy management"/ OR "dose reduction".ti,ab OR "taper*".ti,ab OR exp "drug withdrawal"/ OR exp "deprescription"/ OR exp "inappropriate prescribing"/ OR "appropriate".ti,ab OR "appropriat*".ti,ab OR "inappropriate".ti,ab OR "inappropriat*".ti,ab) AND (exp *"General Practitioner"/ OR "General Practitioner".ti,ab OR "General Practitioners".ti,ab OR "General Practice physician".ti,ab OR "General Practice physicians".ti,ab OR exp **"General Practice"/ OR "General Practice".ti,ab OR "Family Physician".ti,ab OR "Family Physicians".ti,ab OR "Family Practice".ti,ab OR "Primary Care Physician".ti,ab OR "Primary Care Physicians".ti,ab OR exp *"Primary Health Care"/ OR "Primary Care".ti,ab OR "Primary Health Care".ti,ab OR "Primary Healthcare".ti,ab OR "Health Care Personnel"/ OR "Health Care Providers".ti,ab OR "Health Care Provider".ti,ab OR "Healthcare Providers".ti,ab OR "Healthcare Provider".ti,ab OR "Healthcare Workers".ti,ab OR "Healthcare Worker".ti,ab OR "Health Care Professionals".ti,ab OR "Health Care Professional".ti,ab OR "Healthcare Professionals".ti,ab OR "Healthcare Professional".ti,ab OR exp *"Physician"/ OR "Physicians".ti,ab OR "Physician".ti,ab OR "Physician*".ti,ab OR "practitioner".ti,ab OR "practitioners".ti,ab OR "doctor".ti,ab OR "doctors".ti,ab OR "doctor*".ti,ab OR "gastroenterologist".ti,ab OR "gastroenterologists".ti,ab OR "gastroenterologist*".ti,ab OR "internist".ti,ab OR "internists".ti,ab OR "internist*".ti,ab OR "geriatrician".ti,ab OR "geriatricians".ti,ab OR "nursing home physician".ti,ab OR "nursing home physicians".ti,ab OR "nursing home specialist".ti,ab OR "nursing home specialists".ti,ab OR exp *"Pediatrician"/ OR "pediatrician".ti,ab OR "pediatricians".ti,ab OR "paediatrician".ti,ab OR "paediatricians".ti,ab OR "resident".ti,ab OR "residents".ti,ab OR exp *"Resident"/ OR exp *"Pharmacist"/ OR "pharmacist".ti,ab OR "pharmacists".ti,ab) AND (exp *"Proton Pump Inhibitor"/ OR "Proton Pump Inhibitors".ti,ab OR "Proton Pump Inhibitor".ti,ab OR (("PPI".ti,ab OR "PPIs".ti,ab) AND ("dyspepsia".ti,ab OR "dyspeptic".ti,ab OR "reflux".ti,ab OR "gastric".ti,ab OR "stomach".ti,ab)) OR exp *"Dexlansoprazole"/ OR exp *"Esomeprazole"/ OR exp *"Lansoprazole"/ OR exp *"Omeprazole"/ OR exp *"omeprazole, sodium bicarbonate drug combination"/ OR exp *"Pantoprazole"/ OR exp *"Rabeprazole"/ OR exp *"timoprazole"/ OR "1-(2-methyl-4-methoxyphenyl)-4-((2-hydroxyethyl)amino)-6-trifluoromethoxy-2,3-dihydropyrrolo(3,2-c)quinoline".ti,ab OR "1-(2-methylphenyl)-4-methylamino-6-methyl-2,3-dihydropyrrolo(3,2-c)quinoline".ti,ab OR "2,3-dimethyl-8-(2-ethyl-6-methylbenzylamino)imidazo(1,2-a)pyridine-6-carboxamide".ti,ab OR "2-(2-ethylaminobenzylsulfinyl)-5,6-dimethoxybenzimidazole".ti,ab OR "3-(3-(ethoxycarbonyl)propionyl)-8-methoxy-4-((2-methylphenyl)amino)quinoline".ti,ab OR "3-butyryl-4-(2-methylphenylamino)-8-(2-hydroxyethoxy)quinoline".ti,ab OR "3-butyryl-4-(5R-methylbenzylamino)-8-ethoxy-1,7-naphthyridine".ti,ab OR "8-((2-benzimidazolyl)sulfinylmethyl)-1-ethyl-1,2,3,4-tetrahydroquinoline".ti,ab OR "8-(2-ethyl-6-methylbenzylamino)-3-hydroxymethyl-2-methylimidazo(1,2-a)pyridine-6-carboxamide".ti,ab OR "B 823-10".ti,ab OR "BY 831-78".ti,ab OR "BY 841".ti,ab OR "cassigarol A".ti,ab OR "Dexlansoprazole".ti,ab OR "Esomeprazole".ti,ab OR "ethyl 2-((1H-benzimidazol-2-yl)sulfinylmethyl)-4-dimethylamino-5-pyrimidinecarboxylate".ti,ab OR "Lansoprazole".ti,ab OR "Omeprazole".ti,ab OR "omeprazole, sodium bicarbonate drug combination".ti,ab OR "Pantoprazole".ti,ab OR "Rabeprazole".ti,ab OR "S 1924".ti,ab OR "salvianolic acid A".ti,ab OR "scopadulciol".ti,ab OR "SK^ and F 96079".ti,ab OR "SPI 447".ti,ab OR "T 330".ti,ab OR "timoprazole".ti,ab OR "TY 11345".ti,ab OR "xanthoangelol".ti,ab OR "YJA 20379-1".ti,ab OR "YJA 20379-5".ti,ab OR "YJA 20379-6".ti,ab) AND (english.la OR dutch.la))

4. Web of Science

((TI=("prescribing behavior" OR "prescribing behaviors" OR "prescribing behaviour" OR "prescribing behaviours" OR "prescription behavior" OR "prescription behaviors" OR "prescription behaviour" OR "prescription behaviours" OR "Prescribing Pattern" OR "Prescribing Patterns" OR "Prescription Pattern" OR "Prescription Patterns" OR "Prescribing Adherence" OR "Prescription Adherence" OR "Prescription Non Adherence" OR "Prescription Nonadherence" OR "Prescribing Compliance" OR "Prescription Compliance" OR "Prescription Non Compliance" OR "Prescription Noncompliance" OR "prescribing practice" OR "prescribing practice" OR "prescribing practices" OR "prescription practice" OR "prescription practices" OR "Deprescription" OR "Drug Dose Reduction" OR "Inappropriate Prescribing" OR "Medication Error" OR "Drug Utilization" OR "Prescriptions" OR "tapering" OR "taper*" OR "deprescribing" OR "deprescrib*" OR "deprescription" OR "deprescriptions" OR "deprescrip*" OR (("Drug Utilization" OR "Prescriptions" OR "prescription" OR "prescriptions" OR "prescrip*" OR "prescribing" OR "prescribe" OR "prescrib*" OR "medication" OR "medications" OR "medicat*") AND ("appropriate" OR "inappropriate" OR "appropriat*" OR "inappropriat*" OR "cessation" OR "tapering" OR "taper*" OR "stopping" OR "stop" OR "deprescribing" OR "deprescrib*" OR "deprescription" OR "deprescriptions" OR "deprescrip*" OR "implementation" OR "implement*" OR "deimplementation" OR "deimplement*")) OR "deprescrib*" OR ((review* NEAR/3 medication*)) OR (((medication* OR medicines OR prescribing) NEAR/4 inappropriate)) OR "potentially inappropriate" OR ((reduc* NEAR/5 medication*)) OR "polypharmacy" OR "discontinu*" OR "withdraw*" OR ((reducing NEAR/1 (drug* OR inappropriate OR frid))) OR "polypharmacy" OR "medication therapy management" OR "dose reduction" OR "taper*" OR "drug withdrawal" OR "deprescription" OR "inappropriate prescribing" OR "appropriate" OR "appropriat*" OR "inappropriate" OR "inappropriat*") OR AK=("prescribing behavior" OR "prescribing behaviors" OR "prescribing behaviour" OR "prescribing behaviours" OR "prescription behavior" OR "prescription behaviors" OR "prescription behaviour" OR "prescription behaviours" OR "Prescribing Pattern" OR "Prescribing Patterns" OR "Prescription Pattern" OR "Prescription Patterns" OR "Prescribing Adherence" OR "Prescription Adherence" OR "Prescription Non Adherence" OR "Prescription Nonadherence" OR "Prescribing Compliance" OR "Prescription Compliance" OR "Prescription Non Compliance" OR "Prescription Noncompliance" OR "prescribing practice" OR "prescribing practice" OR "prescribing practices" OR "prescription practice" OR "prescription practices" OR "Deprescription" OR "Drug Dose Reduction" OR "Inappropriate Prescribing" OR "Medication Error" OR "Drug Utilization" OR "Prescriptions" OR "tapering" OR "taper*" OR "deprescribing" OR "deprescrib*" OR "deprescription" OR "deprescriptions" OR "deprescrip*" OR (("Drug Utilization" OR "Prescriptions" OR "prescription" OR "prescriptions" OR "prescrip*" OR "prescribing" OR "prescribe" OR "prescrib*" OR "medication" OR "medications" OR "medicat*") AND ("appropriate" OR "inappropriate" OR "appropriat*" OR "inappropriat*" OR "cessation" OR "tapering" OR "taper*" OR "stopping" OR "stop" OR "deprescribing" OR "deprescrib*" OR "deprescription" OR "deprescriptions" OR "deprescrip*" OR "implementation" OR "implement*" OR "deimplementation" OR "deimplement*")) OR "deprescrib*" OR ((review* NEAR/3 medication*)) OR (((medication* OR medicines OR prescribing) NEAR/4 inappropriate)) OR "potentially inappropriate" OR ((reduc* NEAR/5 medication*)) OR "polypharmacy" OR "discontinu*" OR "withdraw*" OR ((reducing NEAR/1 (drug* OR inappropriate OR frid))) OR "polypharmacy" OR "medication therapy management" OR "dose reduction" OR "taper*" OR "drug withdrawal" OR "deprescription" OR "inappropriate prescribing" OR "appropriate" OR "appropriat*" OR "inappropriate" OR "inappropriat*") OR AB=("prescribing behavior" OR "prescribing behaviors" OR "prescribing behaviour" OR "prescribing behaviours" OR "prescription behavior" OR "prescription behaviors" OR "prescription behaviour" OR "prescription behaviours" OR "Prescribing Pattern" OR "Prescribing Patterns" OR "Prescription Pattern" OR "Prescription Patterns" OR "Prescribing Adherence" OR "Prescription Adherence" OR "Prescription Non Adherence" OR "Prescription Nonadherence" OR "Prescribing Compliance" OR "Prescription Compliance" OR "Prescription Non Compliance" OR "Prescription Noncompliance" OR "prescribing practice" OR "prescribing practice" OR "prescribing practices" OR "prescription practice" OR "prescription practices" OR "Deprescription" OR "Drug Dose Reduction" OR "Inappropriate Prescribing" OR "Medication Error" OR "Drug Utilization" OR "Prescriptions" OR "tapering" OR "taper*" OR "deprescribing" OR "deprescrib*" OR "deprescription" OR "deprescriptions" OR "deprescrip*" OR (("Drug Utilization" OR "Prescriptions" OR "prescription" OR "prescriptions" OR "prescrip*" OR "prescribing" OR "prescribe" OR "prescrib*" OR "medication" OR "medications" OR "medicat*") AND ("appropriate" OR "inappropriate" OR "appropriat*" OR "inappropriat*" OR "cessation" OR "tapering" OR "taper*" OR "stopping" OR "stop" OR "deprescribing" OR "deprescrib*" OR "deprescription" OR "deprescriptions" OR "deprescrip*" OR "implementation" OR "implement*" OR "deimplementation" OR "deimplement*")) OR "deprescrib*" OR ((review* NEAR/3 medication*)) OR (((medication* OR medicines OR prescribing) NEAR/4 inappropriate)) OR "potentially inappropriate" OR ((reduc* NEAR/5 medication*)) OR "polypharmacy" OR "discontinu*" OR "withdraw*" OR ((reducing NEAR/1 (drug* OR inappropriate OR frid))) OR "polypharmacy" OR "medication therapy management" OR "dose reduction" OR "taper*" OR "drug withdrawal" OR "deprescription" OR "inappropriate prescribing" OR "appropriate" OR "appropriat*" OR "inappropriate" OR "inappropriat*")) AND (TI=("General Practitioner" OR "General Practitioner" OR "General Practitioners" OR "General Practice physician" OR "General Practice physicians" OR "General Practice" OR "General Practice" OR "Family Physician" OR "Family Physicians" OR "Family Practice" OR "Primary Care Physician" OR "Primary Care Physicians" OR "Primary Health Care" OR "Primary Care" OR "Primary Health Care" OR "Primary Healthcare" OR "Health Care Personnel" OR "Health Care Providers" OR "Health Care Provider" OR "Healthcare Providers" OR "Healthcare Provider" OR "Healthcare Workers" OR "Healthcare Worker" OR "Health Care Professionals" OR "Health Care Professional" OR "Healthcare Professionals" OR "Healthcare Professional" OR "Physician" OR "Physicians" OR "Physician" OR "Physician*" OR "practitioner" OR "practitioners" OR "doctor" OR "doctors" OR "doctor*" OR "gastroenterologist" OR "gastroenterologists" OR "gastroenterologist*" OR "internist" OR "internists" OR "internist*" OR "geriatrician" OR "geriatricians" OR "nursing home physician" OR "nursing home physicians" OR "nursing home specialist" OR "nursing home specialists" OR "Pediatrician" OR "pediatrician" OR "pediatricians" OR "paediatrician" OR "paediatricians" OR "resident" OR "residents" OR "Resident" OR "Pharmacist" OR "pharmacist" OR "pharmacists") OR AK=("General Practitioner" OR "General Practitioner" OR "General Practitioners" OR "General Practice physician" OR "General Practice physicians" OR "General Practice" OR "General Practice" OR "Family Physician" OR "Family Physicians" OR "Family Practice" OR "Primary Care Physician" OR "Primary Care Physicians" OR "Primary Health Care" OR "Primary Care" OR "Primary Health Care" OR "Primary Healthcare" OR "Health Care Personnel" OR "Health Care Providers" OR "Health Care Provider" OR "Healthcare Providers" OR "Healthcare Provider" OR "Healthcare Workers" OR "Healthcare Worker" OR "Health Care Professionals" OR "Health Care Professional" OR "Healthcare Professionals" OR "Healthcare Professional" OR "Physician" OR "Physicians" OR "Physician" OR "Physician*" OR "practitioner" OR "practitioners" OR "doctor" OR "doctors" OR "doctor*" OR "gastroenterologist" OR "gastroenterologists" OR "gastroenterologist*" OR "internist" OR "internists" OR "internist*" OR "geriatrician" OR "geriatricians" OR "nursing home physician" OR "nursing home physicians" OR "nursing home specialist" OR "nursing home specialists" OR "Pediatrician" OR "pediatrician" OR "pediatricians" OR "paediatrician" OR "paediatricians" OR "resident" OR "residents" OR "Resident" OR "Pharmacist" OR "pharmacist" OR "pharmacists") OR AB=("General Practitioner" OR "General Practitioner" OR "General Practitioners" OR "General Practice physician" OR "General Practice physicians" OR "General Practice" OR "General Practice" OR "Family Physician" OR "Family Physicians" OR "Family Practice" OR "Primary Care Physician" OR "Primary Care Physicians" OR "Primary Health Care" OR "Primary Care" OR "Primary Health Care" OR "Primary Healthcare" OR "Health Care Personnel" OR "Health Care Providers" OR "Health Care Provider" OR "Healthcare Providers" OR "Healthcare Provider" OR "Healthcare Workers" OR "Healthcare Worker" OR "Health Care Professionals" OR "Health Care Professional" OR "Healthcare Professionals" OR "Healthcare Professional" OR "Physician" OR "Physicians" OR "Physician" OR "Physician*" OR "practitioner" OR "practitioners" OR "doctor" OR "doctors" OR "doctor*" OR "gastroenterologist" OR "gastroenterologists" OR "gastroenterologist*" OR "internist" OR "internists" OR "internist*" OR "geriatrician" OR "geriatricians" OR "nursing home physician" OR "nursing home physicians" OR "nursing home specialist" OR "nursing home specialists" OR "Pediatrician" OR "pediatrician" OR "pediatricians" OR "paediatrician" OR "paediatricians" OR "resident" OR "residents" OR "Resident" OR "Pharmacist" OR "pharmacist" OR "pharmacists")) AND (TI=("Proton Pump Inhibitor" OR "Proton Pump Inhibitors" OR "Proton Pump Inhibitor" OR (("PPI" OR "PPIs") AND ("dyspepsia" OR "dyspeptic" OR "reflux" OR "gastric" OR "stomach")) OR "1-(2-methyl-4-methoxyphenyl)-4-((2-hydroxyethyl)amino)-6-trifluoromethoxy-2,3-dihydropyrrolo(3,2-c)quinoline" OR "1-(2-methylphenyl)-4-methylamino-6-methyl-2,3-dihydropyrrolo(3,2-c)quinoline" OR "2,3-dimethyl-8-(2-ethyl-6-methylbenzylamino)imidazo(1,2-a)pyridine-6-carboxamide" OR "2-(2-ethylaminobenzylsulfinyl)-5,6-dimethoxybenzimidazole" OR "3-(3-(ethoxycarbonyl)propionyl)-8-methoxy-4-((2-methylphenyl)amino)quinoline" OR "3-butyryl-4-(2-methylphenylamino)-8-(2-hydroxyethoxy)quinoline" OR "3-butyryl-4-(5R-methylbenzylamino)-8-ethoxy-1,7-naphthyridine" OR "8-((2-benzimidazolyl)sulfinylmethyl)-1-ethyl-1,2,3,4-tetrahydroquinoline" OR "8-(2-ethyl-6-methylbenzylamino)-3-hydroxymethyl-2-methylimidazo(1,2-a)pyridine-6-carboxamide" OR "B 823-10" OR "BY 831-78" OR "BY 841" OR "cassigarol A" OR "Dexlansoprazole" OR "Esomeprazole" OR "ethyl 2-((1H-benzimidazol-2-yl)sulfinylmethyl)-4-dimethylamino-5-pyrimidinecarboxylate" OR "Lansoprazole" OR "Omeprazole" OR "omeprazole, sodium bicarbonate drug combination" OR "Pantoprazole" OR "Rabeprazole" OR "S 1924" OR "salvianolic acid A" OR "scopadulciol" OR "SK^ and F 96079" OR "SPI 447" OR "T 330" OR "timoprazole" OR "TY 11345" OR "xanthoangelol" OR "YJA 20379-1" OR "YJA 20379-5" OR "YJA 20379-6") OR AB=("Proton Pump Inhibitor" OR "Proton Pump Inhibitors" OR "Proton Pump Inhibitor" OR (("PPI" OR "PPIs") AND ("dyspepsia" OR "dyspeptic" OR "reflux" OR "gastric" OR "stomach")) OR "1-(2-methyl-4-methoxyphenyl)-4-((2-hydroxyethyl)amino)-6-trifluoromethoxy-2,3-dihydropyrrolo(3,2-c)quinoline" OR "1-(2-methylphenyl)-4-methylamino-6-methyl-2,3-dihydropyrrolo(3,2-c)quinoline" OR "2,3-dimethyl-8-(2-ethyl-6-methylbenzylamino)imidazo(1,2-a)pyridine-6-carboxamide" OR "2-(2-ethylaminobenzylsulfinyl)-5,6-dimethoxybenzimidazole" OR "3-(3-(ethoxycarbonyl)propionyl)-8-methoxy-4-((2-methylphenyl)amino)quinoline" OR "3-butyryl-4-(2-methylphenylamino)-8-(2-hydroxyethoxy)quinoline" OR "3-butyryl-4-(5R-methylbenzylamino)-8-ethoxy-1,7-naphthyridine" OR "8-((2-benzimidazolyl)sulfinylmethyl)-1-ethyl-1,2,3,4-tetrahydroquinoline" OR "8-(2-ethyl-6-methylbenzylamino)-3-hydroxymethyl-2-methylimidazo(1,2-a)pyridine-6-carboxamide" OR "B 823-10" OR "BY 831-78" OR "BY 841" OR "cassigarol A" OR "Dexlansoprazole" OR "Esomeprazole" OR "ethyl 2-((1H-benzimidazol-2-yl)sulfinylmethyl)-4-dimethylamino-5-pyrimidinecarboxylate" OR "Lansoprazole" OR "Omeprazole" OR "omeprazole, sodium bicarbonate drug combination" OR "Pantoprazole" OR "Rabeprazole" OR "S 1924" OR "salvianolic acid A" OR "scopadulciol" OR "SK^ and F 96079" OR "SPI 447" OR "T 330" OR "timoprazole" OR "TY 11345" OR "xanthoangelol" OR "YJA 20379-1" OR "YJA 20379-5" OR "YJA 20379-6") OR AB=("Proton Pump Inhibitor" OR "Proton Pump Inhibitors" OR "Proton Pump Inhibitor" OR (("PPI" OR "PPIs") AND ("dyspepsia" OR "dyspeptic" OR "reflux" OR "gastric" OR "stomach")) OR "1-(2-methyl-4-methoxyphenyl)-4-((2-hydroxyethyl)amino)-6-trifluoromethoxy-2,3-dihydropyrrolo(3,2-c)quinoline" OR "1-(2-methylphenyl)-4-methylamino-6-methyl-2,3-dihydropyrrolo(3,2-c)quinoline" OR "2,3-dimethyl-8-(2-ethyl-6-methylbenzylamino)imidazo(1,2-a)pyridine-6-carboxamide" OR "2-(2-ethylaminobenzylsulfinyl)-5,6-dimethoxybenzimidazole" OR "3-(3-(ethoxycarbonyl)propionyl)-8-methoxy-4-((2-methylphenyl)amino)quinoline" OR "3-butyryl-4-(2-methylphenylamino)-8-(2-hydroxyethoxy)quinoline" OR "3-butyryl-4-(5R-methylbenzylamino)-8-ethoxy-1,7-naphthyridine" OR "8-((2-benzimidazolyl)sulfinylmethyl)-1-ethyl-1,2,3,4-tetrahydroquinoline" OR "8-(2-ethyl-6-methylbenzylamino)-3-hydroxymethyl-2-methylimidazo(1,2-a)pyridine-6-carboxamide" OR "B 823-10" OR "BY 831-78" OR "BY 841" OR "cassigarol A" OR "Dexlansoprazole" OR "Esomeprazole" OR "ethyl 2-((1H-benzimidazol-2-yl)sulfinylmethyl)-4-dimethylamino-5-pyrimidinecarboxylate" OR "Lansoprazole" OR "Omeprazole" OR "omeprazole, sodium bicarbonate drug combination" OR "Pantoprazole" OR "Rabeprazole" OR "S 1924" OR "salvianolic acid A" OR "scopadulciol" OR "SK^ and F 96079" OR "SPI 447" OR "T 330" OR "timoprazole" OR "TY 11345" OR "xanthoangelol" OR "YJA 20379-1" OR "YJA 20379-5" OR "YJA 20379-6")) AND la=(english OR dutch))

5. Cochrane Library

(("prescribing behavior" OR "prescribing behaviors" OR "prescribing behaviour" OR "prescribing behaviours" OR "prescription behavior" OR "prescription behaviors" OR "prescription behaviour" OR "prescription behaviours" OR "Prescribing Pattern" OR "Prescribing Patterns" OR "Prescription Pattern" OR "Prescription Patterns" OR "Prescribing Adherence" OR "Prescription Adherence" OR "Prescription Non Adherence" OR "Prescription Nonadherence" OR "Prescribing Compliance" OR "Prescription Compliance" OR "Prescription Non Compliance" OR "Prescription Noncompliance" OR "prescribing practice" OR "prescribing practice" OR "prescribing practices" OR "prescription practice" OR "prescription practices" OR "Deprescription" OR "Drug Dose Reduction" OR "Inappropriate Prescribing" OR "Medication Error" OR "Drug Utilization" OR "Prescriptions" OR "tapering" OR "taper*" OR "deprescribing" OR "deprescrib*" OR "deprescription" OR "deprescriptions" OR "deprescrip*" OR (("Drug Utilization" OR "Prescriptions" OR "prescription" OR "prescriptions" OR "prescrip*" OR "prescribing" OR "prescribe" OR "prescrib*" OR "medication" OR "medications" OR "medicat*") AND ("appropriate" OR "inappropriate" OR "appropriat*" OR "inappropriat*" OR "cessation" OR "tapering" OR "taper*" OR "stopping" OR "stop" OR "deprescribing" OR "deprescrib*" OR "deprescription" OR "deprescriptions" OR "deprescrip*" OR "implementation" OR "implement*" OR "deimplementation" OR "deimplement*")) OR "deprescrib*" OR ((review* NEAR/3 medication*)) OR (((medication* OR medicines OR prescribing) NEAR/4 inappropriate)) OR "potentially inappropriate" OR ((reduc* NEAR/5 medication*)) OR "polypharmacy" OR "discontinu*" OR "withdraw*" OR ((reducing NEAR/1 (drug* OR inappropriate OR frid))) OR "polypharmacy" OR "medication therapy management" OR "dose reduction" OR "taper*" OR "drug withdrawal" OR "deprescription" OR "inappropriate prescribing" OR "appropriate" OR "appropriat*" OR "inappropriate" OR "inappropriat*") AND ("General Practitioner" OR "General Practitioner" OR "General Practitioners" OR "General Practice physician" OR "General Practice physicians" OR "General Practice" OR "General Practice" OR "Family Physician" OR "Family Physicians" OR "Family Practice" OR "Primary Care Physician" OR "Primary Care Physicians" OR "Primary Health Care" OR "Primary Care" OR "Primary Health Care" OR "Primary Healthcare" OR "Health Care Personnel" OR "Health Care Providers" OR "Health Care Provider" OR "Healthcare Providers" OR "Healthcare Provider" OR "Healthcare Workers" OR "Healthcare Worker" OR "Health Care Professionals" OR "Health Care Professional" OR "Healthcare Professionals" OR "Healthcare Professional" OR "Physician" OR "Physicians" OR "Physician" OR "Physician*" OR "practitioner" OR "practitioners" OR "doctor" OR "doctors" OR "doctor*" OR "gastroenterologist" OR "gastroenterologists" OR "gastroenterologist*" OR "internist" OR "internists" OR "internist*" OR "geriatrician" OR "geriatricians" OR "nursing home physician" OR "nursing home physicians" OR "nursing home specialist" OR "nursing home specialists" OR "Pediatrician" OR "pediatrician" OR "pediatricians" OR "paediatrician" OR "paediatricians" OR "resident" OR "residents" OR "Resident" OR "Pharmacist" OR "pharmacist" OR "pharmacists") AND ("Proton Pump Inhibitor" OR "Proton Pump Inhibitors" OR "Proton Pump Inhibitor" OR (("PPI" OR "PPIs") AND ("dyspepsia" OR "dyspeptic" OR "reflux" OR "gastric" OR "stomach")) OR "1 (2 methyl 4 methoxyphenyl) 4 ((2 hydroxyethyl)amino) 6 trifluoromethoxy 2,3 dihydropyrrolo(3,2 c)quinoline" OR "1 (2 methylphenyl) 4 methylamino 6 methyl 2,3 dihydropyrrolo(3,2 c)quinoline" OR "2,3 dimethyl 8 (2 ethyl 6 methylbenzylamino)imidazo(1,2 a)pyridine 6 carboxamide" OR "2 (2 ethylaminobenzylsulfinyl) 5,6 dimethoxybenzimidazole" OR "3 (3 (ethoxycarbonyl)propionyl) 8 methoxy 4 ((2 methylphenyl)amino)quinoline" OR "3 butyryl 4 (2 methylphenylamino) 8 (2 hydroxyethoxy)quinoline" OR "3 butyryl 4 (5R methylbenzylamino) 8 ethoxy 1,7 naphthyridine" OR "8 ((2 benzimidazolyl)sulfinylmethyl) 1 ethyl 1,2,3,4 tetrahydroquinoline" OR "8 (2 ethyl 6 methylbenzylamino) 3 hydroxymethyl 2 methylimidazo(1,2 a)pyridine 6 carboxamide" OR "B 823 10" OR "BY 831 78" OR "BY 841" OR "cassigarol A" OR "Dexlansoprazole" OR "Esomeprazole" OR "ethyl 2 ((1H benzimidazol 2 yl)sulfinylmethyl) 4 dimethylamino 5 pyrimidinecarboxylate" OR "Lansoprazole" OR "Omeprazole" OR "omeprazole, sodium bicarbonate drug combination" OR "Pantoprazole" OR "Rabeprazole" OR "S 1924" OR "salvianolic acid A" OR "scopadulciol" OR "SK^ and F 96079" OR "SPI 447" OR "T 330" OR "timoprazole" OR "TY 11345" OR "xanthoangelol" OR "YJA 20379 1" OR "YJA 20379 5" OR "YJA 20379 6")):ti,ab,kw

6. Emcare (OVID)

(("prescribing behavior".ti,ab OR "prescribing behaviors".ti,ab OR "prescribing behaviour".ti,ab OR "prescribing behaviours".ti,ab OR "prescription behavior".ti,ab OR "prescription behaviors".ti,ab OR "prescription behaviour".ti,ab OR "prescription behaviours".ti,ab OR "Prescribing Pattern".ti,ab OR "Prescribing Patterns".ti,ab OR "Prescription Pattern".ti,ab OR "Prescription Patterns".ti,ab OR "Prescribing Adherence".ti,ab OR "Prescription Adherence".ti,ab OR "Prescription Non Adherence".ti,ab OR "Prescription Nonadherence".ti,ab OR "Prescribing Compliance".ti,ab OR "Prescription Compliance".ti,ab OR "Prescription Non Compliance".ti,ab OR "Prescription Noncompliance".ti,ab OR exp *"prescribing practice"/ OR "prescribing practice".ti,ab OR "prescribing practices".ti,ab OR "prescription practice".ti,ab OR "prescription practices".ti,ab OR exp *"Deprescription"/ OR exp "Drug Dose Reduction"/ OR exp *"Inappropriate Prescribing"/ OR exp *"Medication Error"/ OR exp *"Drug Utilization"/ OR exp *"Prescriptions"/ OR "tapering".ti,ab OR "taper*".ti,ab OR "deprescribing".ti,ab OR "deprescrib*".ti,ab OR "deprescription".ti,ab OR "deprescriptions".ti,ab OR "deprescrip*".ti,ab OR ((exp *"Drug Utilization"/ OR exp *"Prescriptions"/ OR "prescription".ti,ab OR "prescriptions".ti,ab OR "prescrip*".ti,ab OR "prescribing".ti,ab OR "prescribe".ti,ab OR "prescrib*".ti,ab OR "medication".ti,ab OR "medications".ti,ab OR "medicat*".ti,ab) AND ("appropriate".ti,ab OR "inappropriate".ti,ab OR "appropriat*".ti,ab OR "inappropriat*".ti,ab OR "cessation".ti,ab OR "tapering".ti,ab OR "taper*".ti,ab OR "stopping".ti,ab OR "stop".ti,ab OR "deprescribing".ti,ab OR "deprescrib*".ti,ab OR "deprescription".ti,ab OR "deprescriptions".ti,ab OR "deprescrip*".ti,ab OR "implementation".ti,ab OR "implement*".ti,ab OR "deimplementation".ti,ab OR "deimplement*".ti,ab)) OR "deprescrib*".ti,ab OR ((review* ADJ3 medication*).ti,ab) OR (((medication* OR medicines OR prescribing) ADJ4 inappropriate).ti,ab) OR "potentially inappropriate".ti,ab OR ((reduc* ADJ5 medication*).ti,ab) OR "polypharmacy".ti,ab OR "discontinu*".ti,ab OR "withdraw*".ti,ab OR ((reducing ADJ1 (drug* OR inappropriate OR frid)).ti,ab) OR exp "polypharmacy"/ OR exp "medication therapy management"/ OR "dose reduction".ti,ab OR "taper*".ti,ab OR exp "drug withdrawal"/ OR exp "deprescription"/ OR exp "inappropriate prescribing"/ OR "appropriate".ti,ab OR "appropriat*".ti,ab OR "inappropriate".ti,ab OR "inappropriat*".ti,ab) AND (exp *"General Practitioner"/ OR "General Practitioner".ti,ab OR "General Practitioners".ti,ab OR "General Practice physician".ti,ab OR "General Practice physicians".ti,ab OR exp **"General Practice"/ OR "General Practice".ti,ab OR "Family Physician".ti,ab OR "Family Physicians".ti,ab OR "Family Practice".ti,ab OR "Primary Care Physician".ti,ab OR "Primary Care Physicians".ti,ab OR exp *"Primary Health Care"/ OR "Primary Care".ti,ab OR "Primary Health Care".ti,ab OR "Primary Healthcare".ti,ab OR "Health Care Personnel"/ OR "Health Care Providers".ti,ab OR "Health Care Provider".ti,ab OR "Healthcare Providers".ti,ab OR "Healthcare Provider".ti,ab OR "Healthcare Workers".ti,ab OR "Healthcare Worker".ti,ab OR "Health Care Professionals".ti,ab OR "Health Care Professional".ti,ab OR "Healthcare Professionals".ti,ab OR "Healthcare Professional".ti,ab OR exp *"Physician"/ OR "Physicians".ti,ab OR "Physician".ti,ab OR "Physician*".ti,ab OR "practitioner".ti,ab OR "practitioners".ti,ab OR "doctor".ti,ab OR "doctors".ti,ab OR "doctor*".ti,ab OR "gastroenterologist".ti,ab OR "gastroenterologists".ti,ab OR "gastroenterologist*".ti,ab OR "internist".ti,ab OR "internists".ti,ab OR "internist*".ti,ab OR "geriatrician".ti,ab OR "geriatricians".ti,ab OR "nursing home physician".ti,ab OR "nursing home physicians".ti,ab OR "nursing home specialist".ti,ab OR "nursing home specialists".ti,ab OR exp *"Pediatrician"/ OR "pediatrician".ti,ab OR "pediatricians".ti,ab OR "paediatrician".ti,ab OR "paediatricians".ti,ab OR "resident".ti,ab OR "residents".ti,ab OR exp *"Resident"/ OR exp *"Pharmacist"/ OR "pharmacist".ti,ab OR "pharmacists".ti,ab) AND (exp *"Proton Pump Inhibitor"/ OR "Proton Pump Inhibitors".ti,ab OR "Proton Pump Inhibitor".ti,ab OR (("PPI".ti,ab OR "PPIs".ti,ab) AND ("dyspepsia".ti,ab OR "dyspeptic".ti,ab OR "reflux".ti,ab OR "gastric".ti,ab OR "stomach".ti,ab)) OR exp *"Dexlansoprazole"/ OR exp *"Esomeprazole"/ OR exp *"Lansoprazole"/ OR exp *"Omeprazole"/ OR exp *"omeprazole, sodium bicarbonate drug combination"/ OR exp *"Pantoprazole"/ OR exp *"Rabeprazole"/ OR exp *"timoprazole"/ OR "1-(2-methyl-4-methoxyphenyl)-4-((2-hydroxyethyl)amino)-6-trifluoromethoxy-2,3-dihydropyrrolo(3,2-c)quinoline".ti,ab OR "1-(2-methylphenyl)-4-methylamino-6-methyl-2,3-dihydropyrrolo(3,2-c)quinoline".ti,ab OR "2,3-dimethyl-8-(2-ethyl-6-methylbenzylamino)imidazo(1,2-a)pyridine-6-carboxamide".ti,ab OR "2-(2-ethylaminobenzylsulfinyl)-5,6-dimethoxybenzimidazole".ti,ab OR "3-(3-(ethoxycarbonyl)propionyl)-8-methoxy-4-((2-methylphenyl)amino)quinoline".ti,ab OR "3-butyryl-4-(2-methylphenylamino)-8-(2-hydroxyethoxy)quinoline".ti,ab OR "3-butyryl-4-(5R-methylbenzylamino)-8-ethoxy-1,7-naphthyridine".ti,ab OR "8-((2-benzimidazolyl)sulfinylmethyl)-1-ethyl-1,2,3,4-tetrahydroquinoline".ti,ab OR "8-(2-ethyl-6-methylbenzylamino)-3-hydroxymethyl-2-methylimidazo(1,2-a)pyridine-6-carboxamide".ti,ab OR "B 823-10".ti,ab OR "BY 831-78".ti,ab OR "BY 841".ti,ab OR "cassigarol A".ti,ab OR "Dexlansoprazole".ti,ab OR "Esomeprazole".ti,ab OR "ethyl 2-((1H-benzimidazol-2-yl)sulfinylmethyl)-4-dimethylamino-5-pyrimidinecarboxylate".ti,ab OR "Lansoprazole".ti,ab OR "Omeprazole".ti,ab OR "omeprazole, sodium bicarbonate drug combination".ti,ab OR "Pantoprazole".ti,ab OR "Rabeprazole".ti,ab OR "S 1924".ti,ab OR "salvianolic acid A".ti,ab OR "scopadulciol".ti,ab OR "SK^ and F 96079".ti,ab OR "SPI 447".ti,ab OR "T 330".ti,ab OR "timoprazole".ti,ab OR "TY 11345".ti,ab OR "xanthoangelol".ti,ab OR "YJA 20379-1".ti,ab OR "YJA 20379-5".ti,ab OR "YJA 20379-6".ti,ab) AND (english.la OR dutch.la))

7. Academic Search Premier (EbscoHOST)

((TI("prescribing behavior" OR "prescribing behaviors" OR "prescribing behaviour" OR "prescribing behaviours" OR "prescription behavior" OR "prescription behaviors" OR "prescription behaviour" OR "prescription behaviours" OR "Prescribing Pattern" OR "Prescribing Patterns" OR "Prescription Pattern" OR "Prescription Patterns" OR "Prescribing Adherence" OR "Prescription Adherence" OR "Prescription Non Adherence" OR "Prescription Nonadherence" OR "Prescribing Compliance" OR "Prescription Compliance" OR "Prescription Non Compliance" OR "Prescription Noncompliance" OR "prescribing practice" OR "prescribing practice" OR "prescribing practices" OR "prescription practice" OR "prescription practices" OR "Deprescription" OR "Drug Dose Reduction" OR "Inappropriate Prescribing" OR "Medication Error" OR "Drug Utilization" OR "Prescriptions" OR "tapering" OR "taper*" OR "deprescribing" OR "deprescrib*" OR "deprescription" OR "deprescriptions" OR "deprescrip*" OR (("Drug Utilization" OR "Prescriptions" OR "prescription" OR "prescriptions" OR "prescrip*" OR "prescribing" OR "prescribe" OR "prescrib*" OR "medication" OR "medications" OR "medicat*") AND ("appropriate" OR "inappropriate" OR "appropriat*" OR "inappropriat*" OR "cessation" OR "tapering" OR "taper*" OR "stopping" OR "stop" OR "deprescribing" OR "deprescrib*" OR "deprescription" OR "deprescriptions" OR "deprescrip*" OR "implementation" OR "implement*" OR "deimplementation" OR "deimplement*")) OR "deprescrib*" OR ((review* NEAR/3 medication*)) OR (((medication* OR medicines OR prescribing) NEAR/4 inappropriate)) OR "potentially inappropriate" OR ((reduc* NEAR/5 medication*)) OR "polypharmacy" OR "discontinu*" OR "withdraw*" OR ((reducing NEAR/1 (drug* OR inappropriate OR frid))) OR "polypharmacy" OR "medication therapy management" OR "dose reduction" OR "taper*" OR "drug withdrawal" OR "deprescription" OR "inappropriate prescribing" OR "appropriate" OR "appropriat*" OR "inappropriate" OR "inappropriat*") OR KW("prescribing behavior" OR "prescribing behaviors" OR "prescribing behaviour" OR "prescribing behaviours" OR "prescription behavior" OR "prescription behaviors" OR "prescription behaviour" OR "prescription behaviours" OR "Prescribing Pattern" OR "Prescribing Patterns" OR "Prescription Pattern" OR "Prescription Patterns" OR "Prescribing Adherence" OR "Prescription Adherence" OR "Prescription Non Adherence" OR "Prescription Nonadherence" OR "Prescribing Compliance" OR "Prescription Compliance" OR "Prescription Non Compliance" OR "Prescription Noncompliance" OR "prescribing practice" OR "prescribing practice" OR "prescribing practices" OR "prescription practice" OR "prescription practices" OR "Deprescription" OR "Drug Dose Reduction" OR "Inappropriate Prescribing" OR "Medication Error" OR "Drug Utilization" OR "Prescriptions" OR "tapering" OR "taper*" OR "deprescribing" OR "deprescrib*" OR "deprescription" OR "deprescriptions" OR "deprescrip*" OR (("Drug Utilization" OR "Prescriptions" OR "prescription" OR "prescriptions" OR "prescrip*" OR "prescribing" OR "prescribe" OR "prescrib*" OR "medication" OR "medications" OR "medicat*") AND ("appropriate" OR "inappropriate" OR "appropriat*" OR "inappropriat*" OR "cessation" OR "tapering" OR "taper*" OR "stopping" OR "stop" OR "deprescribing" OR "deprescrib*" OR "deprescription" OR "deprescriptions" OR "deprescrip*" OR "implementation" OR "implement*" OR "deimplementation" OR "deimplement*")) OR "deprescrib*" OR ((review* NEAR/3 medication*)) OR (((medication* OR medicines OR prescribing) NEAR/4 inappropriate)) OR "potentially inappropriate" OR ((reduc* NEAR/5 medication*)) OR "polypharmacy" OR "discontinu*" OR "withdraw*" OR ((reducing NEAR/1 (drug* OR inappropriate OR frid))) OR "polypharmacy" OR "medication therapy management" OR "dose reduction" OR "taper*" OR "drug withdrawal" OR "deprescription" OR "inappropriate prescribing" OR "appropriate" OR "appropriat*" OR "inappropriate" OR "inappropriat*") OR SU("prescribing behavior" OR "prescribing behaviors" OR "prescribing behaviour" OR "prescribing behaviours" OR "prescription behavior" OR "prescription behaviors" OR "prescription behaviour" OR "prescription behaviours" OR "Prescribing Pattern" OR "Prescribing Patterns" OR "Prescription Pattern" OR "Prescription Patterns" OR "Prescribing Adherence" OR "Prescription Adherence" OR "Prescription Non Adherence" OR "Prescription Nonadherence" OR "Prescribing Compliance" OR "Prescription Compliance" OR "Prescription Non Compliance" OR "Prescription Noncompliance" OR "prescribing practice" OR "prescribing practice" OR "prescribing practices" OR "prescription practice" OR "prescription practices" OR "Deprescription" OR "Drug Dose Reduction" OR "Inappropriate Prescribing" OR "Medication Error" OR "Drug Utilization" OR "Prescriptions" OR "tapering" OR "taper*" OR "deprescribing" OR "deprescrib*" OR "deprescription" OR "deprescriptions" OR "deprescrip*" OR (("Drug Utilization" OR "Prescriptions" OR "prescription" OR "prescriptions" OR "prescrip*" OR "prescribing" OR "prescribe" OR "prescrib*" OR "medication" OR "medications" OR "medicat*") AND ("appropriate" OR "inappropriate" OR "appropriat*" OR "inappropriat*" OR "cessation" OR "tapering" OR "taper*" OR "stopping" OR "stop" OR "deprescribing" OR "deprescrib*" OR "deprescription" OR "deprescriptions" OR "deprescrip*" OR "implementation" OR "implement*" OR "deimplementation" OR "deimplement*")) OR "deprescrib*" OR ((review* NEAR/3 medication*)) OR (((medication* OR medicines OR prescribing) NEAR/4 inappropriate)) OR "potentially inappropriate" OR ((reduc* NEAR/5 medication*)) OR "polypharmacy" OR "discontinu*" OR "withdraw*" OR ((reducing NEAR/1 (drug* OR inappropriate OR frid))) OR "polypharmacy" OR "medication therapy management" OR "dose reduction" OR "taper*" OR "drug withdrawal" OR "deprescription" OR "inappropriate prescribing" OR "appropriate" OR "appropriat*" OR "inappropriate" OR "inappropriat*") OR AB("prescribing behavior" OR "prescribing behaviors" OR "prescribing behaviour" OR "prescribing behaviours" OR "prescription behavior" OR "prescription behaviors" OR "prescription behaviour" OR "prescription behaviours" OR "Prescribing Pattern" OR "Prescribing Patterns" OR "Prescription Pattern" OR "Prescription Patterns" OR "Prescribing Adherence" OR "Prescription Adherence" OR "Prescription Non Adherence" OR "Prescription Nonadherence" OR "Prescribing Compliance" OR "Prescription Compliance" OR "Prescription Non Compliance" OR "Prescription Noncompliance" OR "prescribing practice" OR "prescribing practice" OR "prescribing practices" OR "prescription practice" OR "prescription practices" OR "Deprescription" OR "Drug Dose Reduction" OR "Inappropriate Prescribing" OR "Medication Error" OR "Drug Utilization" OR "Prescriptions" OR "tapering" OR "taper*" OR "deprescribing" OR "deprescrib*" OR "deprescription" OR "deprescriptions" OR "deprescrip*" OR (("Drug Utilization" OR "Prescriptions" OR "prescription" OR "prescriptions" OR "prescrip*" OR "prescribing" OR "prescribe" OR "prescrib*" OR "medication" OR "medications" OR "medicat*") AND ("appropriate" OR "inappropriate" OR "appropriat*" OR "inappropriat*" OR "cessation" OR "tapering" OR "taper*" OR "stopping" OR "stop" OR "deprescribing" OR "deprescrib*" OR "deprescription" OR "deprescriptions" OR "deprescrip*" OR "implementation" OR "implement*" OR "deimplementation" OR "deimplement*")) OR "deprescrib*" OR ((review* NEAR/3 medication*)) OR (((medication* OR medicines OR prescribing) NEAR/4 inappropriate)) OR "potentially inappropriate" OR ((reduc* NEAR/5 medication*)) OR "polypharmacy" OR "discontinu*" OR "withdraw*" OR ((reducing NEAR/1 (drug* OR inappropriate OR frid))) OR "polypharmacy" OR "medication therapy management" OR "dose reduction" OR "taper*" OR "drug withdrawal" OR "deprescription" OR "inappropriate prescribing" OR "appropriate" OR "appropriat*" OR "inappropriate" OR "inappropriat*")) AND (TI("General Practitioner" OR "General Practitioner" OR "General Practitioners" OR "General Practice physician" OR "General Practice physicians" OR "General Practice" OR "General Practice" OR "Family Physician" OR "Family Physicians" OR "Family Practice" OR "Primary Care Physician" OR "Primary Care Physicians" OR "Primary Health Care" OR "Primary Care" OR "Primary Health Care" OR "Primary Healthcare" OR "Health Care Personnel" OR "Health Care Providers" OR "Health Care Provider" OR "Healthcare Providers" OR "Healthcare Provider" OR "Healthcare Workers" OR "Healthcare Worker" OR "Health Care Professionals" OR "Health Care Professional" OR "Healthcare Professionals" OR "Healthcare Professional" OR "Physician" OR "Physicians" OR "Physician" OR "Physician*" OR "practitioner" OR "practitioners" OR "doctor" OR "doctors" OR "doctor*" OR "gastroenterologist" OR "gastroenterologists" OR "gastroenterologist*" OR "internist" OR "internists" OR "internist*" OR "geriatrician" OR "geriatricians" OR "nursing home physician" OR "nursing home physicians" OR "nursing home specialist" OR "nursing home specialists" OR "Pediatrician" OR "pediatrician" OR "pediatricians" OR "paediatrician" OR "paediatricians" OR "resident" OR "residents" OR "Resident" OR "Pharmacist" OR "pharmacist" OR "pharmacists") OR KW("General Practitioner" OR "General Practitioner" OR "General Practitioners" OR "General Practice physician" OR "General Practice physicians" OR "General Practice" OR "General Practice" OR "Family Physician" OR "Family Physicians" OR "Family Practice" OR "Primary Care Physician" OR "Primary Care Physicians" OR "Primary Health Care" OR "Primary Care" OR "Primary Health Care" OR "Primary Healthcare" OR "Health Care Personnel" OR "Health Care Providers" OR "Health Care Provider" OR "Healthcare Providers" OR "Healthcare Provider" OR "Healthcare Workers" OR "Healthcare Worker" OR "Health Care Professionals" OR "Health Care Professional" OR "Healthcare Professionals" OR "Healthcare Professional" OR "Physician" OR "Physicians" OR "Physician" OR "Physician*" OR "practitioner" OR "practitioners" OR "doctor" OR "doctors" OR "doctor*" OR "gastroenterologist" OR "gastroenterologists" OR "gastroenterologist*" OR "internist" OR "internists" OR "internist*" OR "geriatrician" OR "geriatricians" OR "nursing home physician" OR "nursing home physicians" OR "nursing home specialist" OR "nursing home specialists" OR "Pediatrician" OR "pediatrician" OR "pediatricians" OR "paediatrician" OR "paediatricians" OR "resident" OR "residents" OR "Resident" OR "Pharmacist" OR "pharmacist" OR "pharmacists") OR SU("General Practitioner" OR "General Practitioner" OR "General Practitioners" OR "General Practice physician" OR "General Practice physicians" OR "General Practice" OR "General Practice" OR "Family Physician" OR "Family Physicians" OR "Family Practice" OR "Primary Care Physician" OR "Primary Care Physicians" OR "Primary Health Care" OR "Primary Care" OR "Primary Health Care" OR "Primary Healthcare" OR "Health Care Personnel" OR "Health Care Providers" OR "Health Care Provider" OR "Healthcare Providers" OR "Healthcare Provider" OR "Healthcare Workers" OR "Healthcare Worker" OR "Health Care Professionals" OR "Health Care Professional" OR "Healthcare Professionals" OR "Healthcare Professional" OR "Physician" OR "Physicians" OR "Physician" OR "Physician*" OR "practitioner" OR "practitioners" OR "doctor" OR "doctors" OR "doctor*" OR "gastroenterologist" OR "gastroenterologists" OR "gastroenterologist*" OR "internist" OR "internists" OR "internist*" OR "geriatrician" OR "geriatricians" OR "nursing home physician" OR "nursing home physicians" OR "nursing home specialist" OR "nursing home specialists" OR "Pediatrician" OR "pediatrician" OR "pediatricians" OR "paediatrician" OR "paediatricians" OR "resident" OR "residents" OR "Resident" OR "Pharmacist" OR "pharmacist" OR "pharmacists") OR AB("General Practitioner" OR "General Practitioner" OR "General Practitioners" OR "General Practice physician" OR "General Practice physicians" OR "General Practice" OR "General Practice" OR "Family Physician" OR "Family Physicians" OR "Family Practice" OR "Primary Care Physician" OR "Primary Care Physicians" OR "Primary Health Care" OR "Primary Care" OR "Primary Health Care" OR "Primary Healthcare" OR "Health Care Personnel" OR "Health Care Providers" OR "Health Care Provider" OR "Healthcare Providers" OR "Healthcare Provider" OR "Healthcare Workers" OR "Healthcare Worker" OR "Health Care Professionals" OR "Health Care Professional" OR "Healthcare Professionals" OR "Healthcare Professional" OR "Physician" OR "Physicians" OR "Physician" OR "Physician*" OR "practitioner" OR "practitioners" OR "doctor" OR "doctors" OR "doctor*" OR "gastroenterologist" OR "gastroenterologists" OR "gastroenterologist*" OR "internist" OR "internists" OR "internist*" OR "geriatrician" OR "geriatricians" OR "nursing home physician" OR "nursing home physicians" OR "nursing home specialist" OR "nursing home specialists" OR "Pediatrician" OR "pediatrician" OR "pediatricians" OR "paediatrician" OR "paediatricians" OR "resident" OR "residents" OR "Resident" OR "Pharmacist" OR "pharmacist" OR "pharmacists")) AND (TI("Proton Pump Inhibitor" OR "Proton Pump Inhibitors" OR "Proton Pump Inhibitor" OR (("PPI" OR "PPIs") AND ("dyspepsia" OR "dyspeptic" OR "reflux" OR "gastric" OR "stomach")) OR "1-(2-methyl-4-methoxyphenyl)-4-((2-hydroxyethyl)amino)-6-trifluoromethoxy-2,3-dihydropyrrolo(3,2-c)quinoline" OR "1-(2-methylphenyl)-4-methylamino-6-methyl-2,3-dihydropyrrolo(3,2-c)quinoline" OR "2,3-dimethyl-8-(2-ethyl-6-methylbenzylamino)imidazo(1,2-a)pyridine-6-carboxamide" OR "2-(2-ethylaminobenzylsulfinyl)-5,6-dimethoxybenzimidazole" OR "3-(3-(ethoxycarbonyl)propionyl)-8-methoxy-4-((2-methylphenyl)amino)quinoline" OR "3-butyryl-4-(2-methylphenylamino)-8-(2-hydroxyethoxy)quinoline" OR "3-butyryl-4-(5R-methylbenzylamino)-8-ethoxy-1,7-naphthyridine" OR "8-((2-benzimidazolyl)sulfinylmethyl)-1-ethyl-1,2,3,4-tetrahydroquinoline" OR "8-(2-ethyl-6-methylbenzylamino)-3-hydroxymethyl-2-methylimidazo(1,2-a)pyridine-6-carboxamide" OR "B 823-10" OR "BY 831-78" OR "BY 841" OR "cassigarol A" OR "Dexlansoprazole" OR "Esomeprazole" OR "ethyl 2-((1H-benzimidazol-2-yl)sulfinylmethyl)-4-dimethylamino-5-pyrimidinecarboxylate" OR "Lansoprazole" OR "Omeprazole" OR "omeprazole, sodium bicarbonate drug combination" OR "Pantoprazole" OR "Rabeprazole" OR "S 1924" OR "salvianolic acid A" OR "scopadulciol" OR "SK^ and F 96079" OR "SPI 447" OR "T 330" OR "timoprazole" OR "TY 11345" OR "xanthoangelol" OR "YJA 20379-1" OR "YJA 20379-5" OR "YJA 20379-6") OR KW("Proton Pump Inhibitor" OR "Proton Pump Inhibitors" OR "Proton Pump Inhibitor" OR (("PPI" OR "PPIs") AND ("dyspepsia" OR "dyspeptic" OR "reflux" OR "gastric" OR "stomach")) OR "1-(2-methyl-4-methoxyphenyl)-4-((2-hydroxyethyl)amino)-6-trifluoromethoxy-2,3-dihydropyrrolo(3,2-c)quinoline" OR "1-(2-methylphenyl)-4-methylamino-6-methyl-2,3-dihydropyrrolo(3,2-c)quinoline" OR "2,3-dimethyl-8-(2-ethyl-6-methylbenzylamino)imidazo(1,2-a)pyridine-6-carboxamide" OR "2-(2-ethylaminobenzylsulfinyl)-5,6-dimethoxybenzimidazole" OR "3-(3-(ethoxycarbonyl)propionyl)-8-methoxy-4-((2-methylphenyl)amino)quinoline" OR "3-butyryl-4-(2-methylphenylamino)-8-(2-hydroxyethoxy)quinoline" OR "3-butyryl-4-(5R-methylbenzylamino)-8-ethoxy-1,7-naphthyridine" OR "8-((2-benzimidazolyl)sulfinylmethyl)-1-ethyl-1,2,3,4-tetrahydroquinoline" OR "8-(2-ethyl-6-methylbenzylamino)-3-hydroxymethyl-2-methylimidazo(1,2-a)pyridine-6-carboxamide" OR "B 823-10" OR "BY 831-78" OR "BY 841" OR "cassigarol A" OR "Dexlansoprazole" OR "Esomeprazole" OR "ethyl 2-((1H-benzimidazol-2-yl)sulfinylmethyl)-4-dimethylamino-5-pyrimidinecarboxylate" OR "Lansoprazole" OR "Omeprazole" OR "omeprazole, sodium bicarbonate drug combination" OR "Pantoprazole" OR "Rabeprazole" OR "S 1924" OR "salvianolic acid A" OR "scopadulciol" OR "SK^ and F 96079" OR "SPI 447" OR "T 330" OR "timoprazole" OR "TY 11345" OR "xanthoangelol" OR "YJA 20379-1" OR "YJA 20379-5" OR "YJA 20379-6") OR SU("Proton Pump Inhibitor" OR "Proton Pump Inhibitors" OR "Proton Pump Inhibitor" OR (("PPI" OR "PPIs") AND ("dyspepsia" OR "dyspeptic" OR "reflux" OR "gastric" OR "stomach")) OR "1-(2-methyl-4-methoxyphenyl)-4-((2-hydroxyethyl)amino)-6-trifluoromethoxy-2,3-dihydropyrrolo(3,2-c)quinoline" OR "1-(2-methylphenyl)-4-methylamino-6-methyl-2,3-dihydropyrrolo(3,2-c)quinoline" OR "2,3-dimethyl-8-(2-ethyl-6-methylbenzylamino)imidazo(1,2-a)pyridine-6-carboxamide" OR "2-(2-ethylaminobenzylsulfinyl)-5,6-dimethoxybenzimidazole" OR "3-(3-(ethoxycarbonyl)propionyl)-8-methoxy-4-((2-methylphenyl)amino)quinoline" OR "3-butyryl-4-(2-methylphenylamino)-8-(2-hydroxyethoxy)quinoline" OR "3-butyryl-4-(5R-methylbenzylamino)-8-ethoxy-1,7-naphthyridine" OR "8-((2-benzimidazolyl)sulfinylmethyl)-1-ethyl-1,2,3,4-tetrahydroquinoline" OR "8-(2-ethyl-6-methylbenzylamino)-3-hydroxymethyl-2-methylimidazo(1,2-a)pyridine-6-carboxamide" OR "B 823-10" OR "BY 831-78" OR "BY 841" OR "cassigarol A" OR "Dexlansoprazole" OR "Esomeprazole" OR "ethyl 2-((1H-benzimidazol-2-yl)sulfinylmethyl)-4-dimethylamino-5-pyrimidinecarboxylate" OR "Lansoprazole" OR "Omeprazole" OR "omeprazole, sodium bicarbonate drug combination" OR "Pantoprazole" OR "Rabeprazole" OR "S 1924" OR "salvianolic acid A" OR "scopadulciol" OR "SK^ and F 96079" OR "SPI 447" OR "T 330" OR "timoprazole" OR "TY 11345" OR "xanthoangelol" OR "YJA 20379-1" OR "YJA 20379-5" OR "YJA 20379-6") OR AB("Proton Pump Inhibitor" OR "Proton Pump Inhibitors" OR "Proton Pump Inhibitor" OR (("PPI" OR "PPIs") AND ("dyspepsia" OR "dyspeptic" OR "reflux" OR "gastric" OR "stomach")) OR "1-(2-methyl-4-methoxyphenyl)-4-((2-hydroxyethyl)amino)-6-trifluoromethoxy-2,3-dihydropyrrolo(3,2-c)quinoline" OR "1-(2-methylphenyl)-4-methylamino-6-methyl-2,3-dihydropyrrolo(3,2-c)quinoline" OR "2,3-dimethyl-8-(2-ethyl-6-methylbenzylamino)imidazo(1,2-a)pyridine-6-carboxamide" OR "2-(2-ethylaminobenzylsulfinyl)-5,6-dimethoxybenzimidazole" OR "3-(3-(ethoxycarbonyl)propionyl)-8-methoxy-4-((2-methylphenyl)amino)quinoline" OR "3-butyryl-4-(2-methylphenylamino)-8-(2-hydroxyethoxy)quinoline" OR "3-butyryl-4-(5R-methylbenzylamino)-8-ethoxy-1,7-naphthyridine" OR "8-((2-benzimidazolyl)sulfinylmethyl)-1-ethyl-1,2,3,4-tetrahydroquinoline" OR "8-(2-ethyl-6-methylbenzylamino)-3-hydroxymethyl-2-methylimidazo(1,2-a)pyridine-6-carboxamide" OR "B 823-10" OR "BY 831-78" OR "BY 841" OR "cassigarol A" OR "Dexlansoprazole" OR "Esomeprazole" OR "ethyl 2-((1H-benzimidazol-2-yl)sulfinylmethyl)-4-dimethylamino-5-pyrimidinecarboxylate" OR "Lansoprazole" OR "Omeprazole" OR "omeprazole, sodium bicarbonate drug combination" OR "Pantoprazole" OR "Rabeprazole" OR "S 1924" OR "salvianolic acid A" OR "scopadulciol" OR "SK^ and F 96079" OR "SPI 447" OR "T 330" OR "timoprazole" OR "TY 11345" OR "xanthoangelol" OR "YJA 20379-1" OR "YJA 20379-5" OR "YJA 20379-6")))

8. Google Scholar

Twelve permutations - first 20-100 references saved.

"prescribing behavior"|"prescribing behaviors"|"Prescribing Pattern"|"Prescribing Patterns" "Practitioner"|"Practitioners"|"physician"|"physicians"|"doctor"|"doctors" "Proton Pump Inhibitor"|"Proton Pump Inhibitors"|"PPI"|"PPIs"

"prescribing behavior"|"prescribing behaviors"|"Prescribing Pattern"|"Prescribing Patterns" "gastroenterologist"|"gastroenterologists"|"internist"|"internists" "Proton Pump Inhibitor"|"Proton Pump Inhibitors"|"PPI"|"PPIs"

"prescribing behavior"|"prescribing behaviors"|"Prescribing Pattern"|"Prescribing Patterns" "geriatrician"|"geriatricians"|"pediatrician"|"pediatricians" "Proton Pump Inhibitor"|"Proton Pump Inhibitors"|"PPI"|"PPIs"

"prescribing behavior"|"prescribing behaviors"|"Prescribing Pattern"|"Prescribing Patterns" "paediatrician"|"paediatricians"|"resident"|"residents"|"pharmacist"|"pharmacists" "Proton Pump Inhibitor"|"Proton Pump Inhibitors"|"PPI"|"PPIs"

"deprescribing"|"Deprescription"|"deprescriptions" "Practitioner"|"Practitioners"|"physician"|"physicians"|"doctor"|"doctors" "Proton Pump Inhibitor"|"Proton Pump Inhibitors"|"PPI"|"PPIs"

"deprescribing"|"Deprescription"|"deprescriptions" "gastroenterologist"|"gastroenterologists"|"internist"|"internists" "Proton Pump Inhibitor"|"Proton Pump Inhibitors"|"PPI"|"PPIs"

"deprescribing"|"Deprescription"|"deprescriptions" "geriatrician"|"geriatricians"|"pediatrician"|"pediatricians" "Proton Pump Inhibitor"|"Proton Pump Inhibitors"|"PPI"|"PPIs"

"deprescribing"|"Deprescription"|"deprescriptions" "paediatrician"|"paediatricians"|"resident"|"residents"|"pharmacist"|"pharmacists" "Proton Pump Inhibitor"|"Proton Pump Inhibitors"|"PPI"|"PPIs"

"Inappropriate Prescribing"|"Inappropriate Prescription"|"Inappropriate Prescriptions" "Practitioner"|"Practitioners"|"physician"|"physicians"|"doctor"|"doctors" "Proton Pump Inhibitor"|"Proton Pump Inhibitors"|"PPI"|"PPIs"

"Inappropriate Prescribing"|"Inappropriate Prescription"|"Inappropriate Prescriptions" "gastroenterologist"|"gastroenterologists"|"internist"|"internists" "Proton Pump Inhibitor"|"Proton Pump Inhibitors"|"PPI"|"PPIs"

"Inappropriate Prescribing"|"Inappropriate Prescription"|"Inappropriate Prescriptions" "geriatrician"|"geriatricians"|"pediatrician"|"pediatricians" "Proton Pump Inhibitor"|"Proton Pump Inhibitors"|"PPI"|"PPIs"

"Inappropriate Prescribing"|"Inappropriate Prescription"|"Inappropriate Prescriptions" "paediatrician"|"paediatricians"|"resident"|"residents"|"pharmacist"|"pharmacists" "Proton Pump Inhibitor"|"Proton Pump Inhibitors"|"PPI"|"PPIs"
