# Supplementary material for: Determinants of and interventions for Proton Pump Inhibitor prescription behavior: A systematic scoping review
Source: BMC Prim Care. 2024 Jun 11;25:208. doi: 10.1186/s12875-024-02459-5 (PMC11165893; doi:10.1186/s12875-024-02459-5)
Supplement: Supplementary file 4 — Supplementary Material 4. [file 12875_2024_2459_MOESM4_ESM.pdf]

Determinants of proton pump inhibitor prescription behavior: a systematic scoping review protocol

24-06-2022

G. van den Brink[1,2]
L.C. van Gestel[3]
S.M. Mensink-Bout[1,2]
M. Adriaanse[3]
J.C. Kiefte-de Jong[1,2]

[1] Department of Public Health and Primary Care, Leiden University Medical Center
[2] Health Campus The Hague
[3] Faculty of Social and Behavioural Sciences, Leiden University

STUDY INFORMATION

Background

Proton pump inhibitors (PPIs) are effective in the treatment of gastric symptoms such as dyspepsia or reflux, but chronic use has been related to serious side effects [1]. Patients that visit their general practitioner in the Netherlands with dyspepsia without alarm symptoms receive advice to adapt lifestyle aspects that are known to affect dyspepsia, including some dietary factors, overweight and smoking [2-4]. If lifestyle changes are not sufficient, patients can start medical therapy with a step up approach, in which PPIs are the third step [5]. In patients with a short term PPI indication, discontinuation within 3 months is recommended because of the association of chronic PPI use with an increased risk of bacterial infections, bone fractures, vitamin B12 and iron deficiency and an impaired kidney function [6]. However, primary care studies show that in the Netherlands half of the PPI users lack a valid indication and that 30-60% of patients use PPIs longer than the advised 3 months [2]. Similar numbers have been observed in other high income countries [7-9]. So, PPIs are often prescribed without the right indication or too long. This inappropriate PPI prescribing urgently needs to be addressed.

Deprescribing refers to the process of tapering or withdrawing of medications that lack a valid indication under the supervision of a health care professional [10]. An important stakeholder in deprescribing PPIs is the prescribing provider. However, to our knowledge, previous studies investigating deprescribing PPIs did not take this behavioral science perspective into account. A comprehensive picture of the behavioral determinants of PPI prescription in primary care is required to allow for designing effective interventions that

include that include those Behavior Change Techniques (BCTs) that target the most important and changeable determinants of this behavior. When prescription behavior is target of a behavioral intervention, providers will be stimulated to follow the current guidelines and prescribe less PPIs, which will subsequently lower inappropriate PPI use.

Aim

Our aim is to perform a systematic scoping literature review identifying determinants of PPI prescription behavior.

Review question

What are determinants of PPI prescription behavior?

STUDY DESIGN PLAN

We will use a scoping review to evaluate our research question. This method is suitable to evaluate and summarize the body of evidence which is heterogeneous in methods or discipline [11]. The scoping review will be performed according to the recommendations of the Joanna Briggs Institute guidance [12], which is based on earlier recommendations [13, 14]. We will report the results of our review according to the PRISMA-ScR (Preferred Reporting Items for Systematic reviews and Meta-Analyses extension for Scoping Reviews) recommendations [11].

Data collection procedures

A literature search will be conducted in which the primary research question is addressed. The search will be defined in consultation with an information specialist from the Leiden University Medical Center. The principal reviewers will provide key articles from previous literature and corresponding keywords. We will perform an additional search in the grey literature for unpublished studies or studies published by commercial publishers. After full text screening, we will use citation tracking to identify potential additional studies.

The following databases will be searched:

- PubMed
- MEDLINE (OVID
- Embase (OVID)
- Web of Science
- Cochrane Library
- Emcare (OVID)
- Academic Search Premier (EbscoHOST)

- Google Scholar

The followinig grey literature databases will be searched:

- Google

Inclusion

- We will include all studies that evaluate determinants of physicians prescribing behavior with regard to PPIs
- We will include all qualitative and quantitative study designs
- We will include all published articles until the search date

Exclusion

- We will exclude articles written in another language than Dutch or English

Study selection

The screening process will be conducted using ASReview (Automatic Systematic Reviews) (v1.0) [15]. ASReview is an active-learning-based software program which presents the papers to the reviewer in an order based on relevance. All papers identified by our search will be imported into ASReview. We will subsequently, based on our research question, identify 3 relevant and 3 irrelevant papers, after which ASReview reorders the papers. Two reviewers will independently screen the reordered papers, starting with the most relevant papers. The papers will be screened on title and abstract to match the inclusion and exclusion criteria. If no abstract is available, a full-text review will be performed. It has previously been shown that by using ASReview 95% of the eligible studies can be identified after screening 8% to 33% of the studies [15]. Therefore, when at least 33% of all the papers found in the search are screened AND when ASReview gives 25 consecutive non-relevant papers, we will stop the screening of the studies. Because the papers are ordered by ASReview based on relevance, we expect that the papers that are still in the dataset at this point can be excluded.

After the initial screening, the results of the included studies will be compared between the two reviewers. Any disagreement will be discussed with a third reviewer. After consensus is found on all papers, the full text articles will be downloaded. If after reading the full text the article appears not to match the inclusion criteria, it will be excluded if both reviewers agree. Any discrepancies at this stage will be discussed and a third reviewer will be consulted to determine final inclusion.

Data extraction

Data will be charted using a data charting form [11, 12]. A predefined form will be jointly developed by two reviewers. Data will be extracted by one reviewer and verified by a second reviewer. Consensus over discrepancies will be reached through discussion or a third reviewer will be consulted. At least the following data will be extracted, as previously recommended [12]:

- Author(s)
- Publication year
- Country of origin
- Aims
- Concept or approach (i.e. qualitative / quantitative)
- Methodology
- Study population and sample size (if applicable)
- Type of determinant
- Intervention type and comparator (if applicable)
- Duration of the intervention (if applicable)
- Measurement of outcomes
- Key findings that relate to the review question

If needed, data extraction form will be continuously updated during the data charting process.

Collating, summarizing and reporting the results

The scoping review will be written in English, and according to the PRISMA-ScR checklist [11]. Results from the chartered data will be summarized based on the type and content of de included studies. A narrative general interpretation will be provided which addresses the research question, as well as the potential implications and directions for future research.

REFERENCES

1. Haastrup, P.F., et al., *Side Effects of Long-Term Proton Pump Inhibitor Use: A Review.* Basic Clin Pharmacol Toxicol, 2018. **123**(2): p. 114-121.
2. De Jongh, E., et al., *NHG-Standaard Maagklachten*, in *(vijfde herziening)*. 2021, Utrecht.
3. Ness-Jensen, E., et al., *Lifestyle Intervention in Gastroesophageal Reflux Disease.* Clin Gastroenterol Hepatol, 2016. **14**(2): p. 175-82 e1-3.
4. Ness-Jensen, E. and J. Lagergren, *Tobacco smoking, alcohol consumption and gastro-oesophageal reflux disease.* Best Pract Res Clin Gastroenterol, 2017. **31**(5): p. 501-508.

5. van Marrewijk, C.J., et al., *Effect and cost-effectiveness of step-up versus step-down treatment with antacids, H2-receptor antagonists, and proton pump inhibitors in patients with new onset dyspepsia (DIAMOND study): a primary-care-based randomised controlled trial.* Lancet, 2009. **373**(9659): p. 215-25.
6. Targownik, L., *Discontinuing Long-Term PPI Therapy: Why, With Whom, and How?* Am J Gastroenterol, 2018. **113**(4): p. 519-528.
7. Lassalle, M., et al., *Use of proton pump inhibitors in adults in France: a nationwide drug utilization study.* Eur J Clin Pharmacol, 2020. **76**(3): p. 449-457.
8. Batuwitage, B.T., et al., *Inappropriate prescribing of proton pump inhibitors in primary care.* Postgrad Med J, 2007. **83**(975): p. 66-8.
9. Lanas, A., *We Are Using Too Many PPIs, and We Need to Stop: A European Perspective.* Am J Gastroenterol, 2016. **111**(8): p. 1085-6.
10. Morel, T., et al., *Development and validation of search filters to identify articles on deprescribing in Medline and Embase.* BMC Med Res Methodol, 2022. **22**(1): p. 79.
11. Tricco, A.C., et al., *PRISMA Extension for Scoping Reviews (PRISMA-ScR): Checklist and Explanation.* Ann Intern Med, 2018. **169**(7): p. 467-473.
12. Peters, M.D., et al., *Guidance for conducting systematic scoping reviews.* Int J Evid Based Healthc, 2015. **13**(3): p. 141-6.
13. Arksey, H. and L. O'Malley, *Scoping studies: towards a methodological framework.* International Journal of Social Research Methodology, 2005. **8**: p. 19-32.
14. Levac, D., H. Colquhoun, and K.K. O'Brien, *Scoping studies: advancing the methodology.* Implement Sci, 2010. **5**: p. 69.
15. van de Schoot, R., et al., *An open source machine learning framework for efficient and transparent systematic reviews.* Nature Machine Intelligence, 2021. **3**(2): p. 125-133.
